# In Combo Studies for the Optimization of 5-Aminoanthranilic Acid Derivatives as Potential Multitarget Drugs for the Management of Metabolic Syndrome

**DOI:** 10.3390/ph15121461

**Published:** 2022-11-25

**Authors:** Edwin Chávez-Gutiérrez, Matilda Martínez-Arellanes, Montserrat Murillo-López, María Fernanda Medina-Guzmán, Laila Mobarak-Richaud, Karen Pelcastre-Guzmán, Osvaldo Javier Quintana-Romero, Armando Ariza-Castolo, María del Rosario Ayala-Moreno, Juan Rodrigo Salazar, Christian Guerra-Araiza, Lorena Rodríguez-Páez, Rodolfo Pinto-Almazán, Marco A. Loza-Mejía

**Affiliations:** 1 Design, Isolation and Synthesis of Bioactive Molecules Research Group, Universidad La Salle-México, Benjamín Franklin 45, Mexico City 06140, Mexico; 2 Doctorado en Ciencias en Biomedicina y Biotecnología Molecular, Escuela Nacional de Ciencias Biológicas, Instituto Politécnico Nacional, Prolongación Manuel Carpio y Plan de Ayala s/n, Mexico City 11340, Mexico; 3 Department of Chemistry, Center for Research and Advanced Studies, The National Polytechnic Institute (CINVESTAV-IPN), Av. Instituto Politécnico Nacional 2508, Mexico City 07360, Mexico; 4 Noncommunicable Diseases Research Group, Universidad La Salle-México, Benjamín Franklin 45, Mexico City 06140, Mexico; 5 Medical Research Unit in Pharmacology, Specialities Hospital Bernardo Sepúlveda, National Medical Center XXI Century, Social Security Mexican Institute (IMSS), Av. Cuauhtémoc 330, Mexico City 06720, Mexico; 6 Biochemistry Department, Escuela Nacional de Ciencias Biológicas, Instituto Politécnico Nacional, Prolongación Manuel Carpio y Plan de Ayala s/n, Mexico City 11340, Mexico; 7 Sección de Estudios de Posgrado e Investigación, Escuela Superior de Medicina, Instituto Politécnico Nacional, Plan de San Luis y Díaz Mirón, Mexico City 11340, Mexico

**Keywords:** metabolic syndrome, multitarget drugs, molecular docking, anthranilic acid

## Abstract

Metabolic syndrome is a set of risk factors that consist of abdominal obesity, arterial hypertension, alterations in the lipid profile, and hyperglycemia. The current therapeutic strategy includes polypharmacy, using three or more drugs to control each syndrome component. However, this approach has drawbacks that could lead to therapeutic failure. Multitarget drugs are molecules with the ability to act on different targets simultaneously and are an attractive alternative for treating complex diseases such as metabolic syndrome. Previously, we identified a triamide derivative of 5-aminoanthranilic acid that exhibited hypoglycemic, hypolipemic, and antihypertensive activities simultaneously. In the present study, we report the synthesis and in combo evaluation of new derivatives of anthranilic acid, intending to identify the primary structural factors that improve the activity over metabolic syndrome-related parameters. We found that substitution on position 5, incorporation of 3,4-dimethoxyphenyl substituents, and having a free carboxylic acid group lead to the in vitro inhibition of HMG-CoA reductase, and simultaneously the diminution of the serum levels of glucose, triglycerides, and cholesterol in a diet-induced in vivo model.

## 1. Introduction

**Figure 1:**
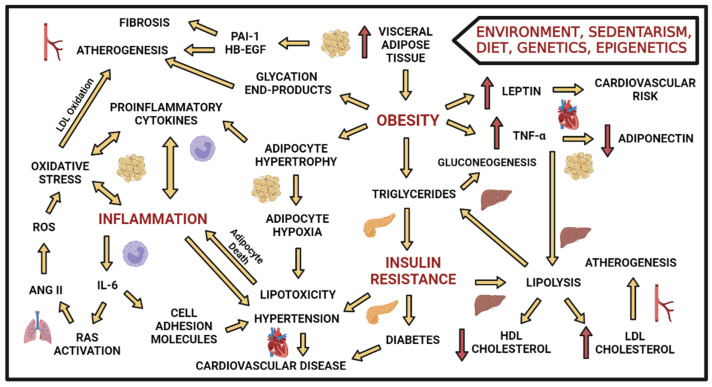
Resumed pathophysiology of metabolic syndrome.

Nowadays, metabolic diseases are one of the most critical health issues worldwide. Metabolic syndrome (MetS) is an assortment of risk factors that cluster abdominal obesity, hypertension, alterations in the lipid profile, and hyperglycemia and is associated with other comorbidities such as prothrombotic state, nonalcoholic fatty liver disease (NAFLD), and reproductive disorders [1,2]. It is estimated that 20–25% of the world’s adult population suffers from MetS, leading to an increased risk of all-cause mortality, especially from cardiovascular diseases [3]. Also, it increases the risk of mortality by infectious agents, as was evidenced during the COVID-19 pandemic [4,5]. Factors that raise the likelihood of developing MetS are genetic background, hypercaloric diet intake, sedentarism, malnutrition, and body habits [2,6,7]. The pathophysiology of MetS consists of complex mechanisms, of which there are still pathways that have not been fully elucidated. Furthermore, it is still under debate whether the individual components of MetS should be treated as distinct pathologies or as manifestations of a common pathogenic mechanism, which is resumed in Figure 1 [8,9]. Of the proposed mechanisms, insulin resistance, neurohormonal activation, and chronic inflammation are the main dysregulated processes involved in the onset and development of MetS and its transition to cardiovascular disease.

The primary intervention in MetS treatment is lifestyle modification, mainly by increased physical activity and dietary change. The focus of such strategies is weight reduction and the control of metabolic parameters [1,10]. However, a pharmacological intervention for MetS is required in more advanced cases. Currently, there is no single-drug therapy for MetS. Consequently, the current pharmacotherapy focus is on the individual management of each metabolic abnormality and associated comorbidities, resulting in the necessity of polypharmacy, primarily as hypoglycemic and hypotensive drugs, statins for dyslipidemia treatment, and antiplatelet drugs to decrease prothrombotic risk [11]. However, polypharmacy tends to increase the risk of adverse outcomes due to drug–drug interactions or medication errors, prescribing cascade, duplication of therapies, and lack of treatment adherence, alongside an increase in the patient’s financial burden [12,13,14,15].

Indeed, one of the current challenges in Medicinal Chemistry is the development of successful drugs to treat multifactorial diseases such as MetS. The traditional “single-target” approach, which is focused on the development of ligands with high selectivity to a single biological entity (“on-target”), has clear advantages. First, it reduces the probability of undesirable effects resulting from interaction with other unwanted biological targets (“off-targets”). Second, the expected therapeutical results can be explained and modulated if needed [16]. However, the complexity of multifactorial pathologies suggests that a single-target approach may be insufficient. In this challenging scenario, multitarget drugs seem an attractive option: the synergism of the simultaneous modulation of two or more targets is more effective in illnesses where multiple pathways are involved in the onset and progression of the disease [17,18].

A multitarget drug can be defined as a drug that modulates multiple targets simultaneously [19,20,21]. This therapeutic approach offers some advantages over the traditional “singlet-target” approximation. It has been described that they exhibit higher therapeutic effects, simpler administration, less probability of drug−drug interaction, and the reduction of the development of drug resistance [22,23,24]. One of the most used strategies for multitarget drug design is to select a privileged scaffold from natural or synthetic origin, followed by optimization of this initial structure, usually guided by computational tools [25,26,27,28,29,30]. To confirm these in silico predictions, further in vitro and in vivo evaluation is required resulting in authentic in combo studies [31,32,33,34,35,36,37]. 

**Figure 2:**
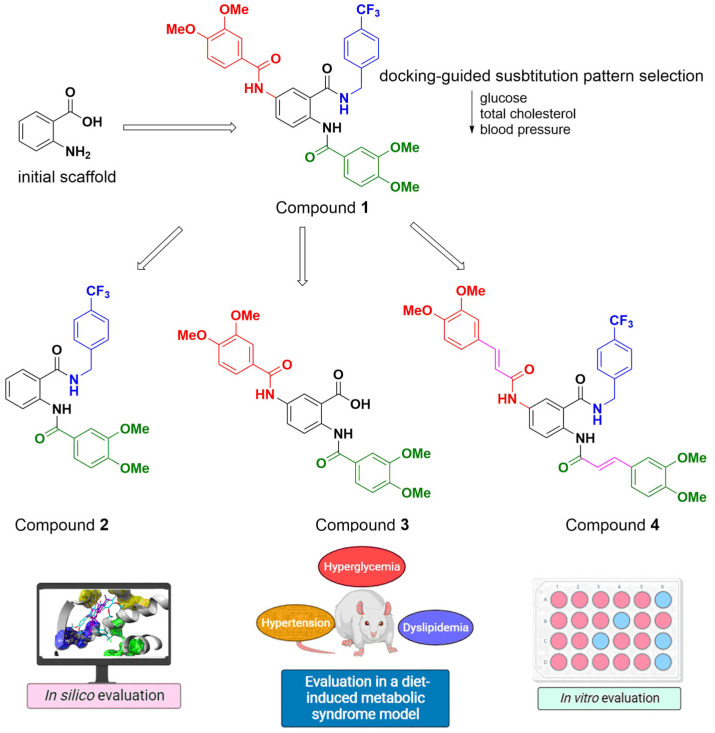
Modifications of compound **1** evaluated in this work.

In our case, we selected anthranilic acid as the privileged scaffold. Previously, we reported the design, synthesis, and evaluation of compound **1** (Figure 2), a triamide derivative of 5-aminoanthranilic acid, as a potential multitarget drug for managing MetS [38]. The design of this compound was based on anthranilic acid as the initial template, since it is a privileged scaffold included as the core of compounds that have exhibited several bioactivities, including good binding properties against some targets related to metabolic diseases [39,40,41,42,43,44,45]. The incorporation of the appropriate substituents that could increase the affinity against PPAR-α, PPAR-γ, HMG-CoA reductase, and angiotensin-converting enzyme (ACE) was directed through molecular docking.

Compound **1** simultaneously diminished the glucose, triglyceride, total cholesterol serum levels, and blood pressure in an in vivo diet-induced MetS model [46,47]. This holistic model offers the advantage of providing relevant information on several bioactivities, allowing quick-go or no-go decisions and reducing the number of animals necessary to demonstrate multiple therapeutic effects [48,49].

To identify the structural factors related to the effect of substituted 5-aminoanthranilic acid derivatives over the parameters of MetS, we decided to modify the structure of compound **1**, as depicted in Figure 2. These modifications included the simplification of the structure of compound **1** to render compounds **2** and **3**, which could improve the physicochemical properties associated with the ADME profile since these compounds would not violate any of Lipinski’s rules; in addition, their bioevaluation would clarify the most relevant structural factors related to their multitarget properties. Additionally, we proposed compound **4** since it has been reported that incorporating ferulic acid-like moieties improves antioxidant and cardioprotective activities, and ferulic acid itself exhibited positive effects in a MetS rodent model [50,51,52,53]. In this work, we present the synthesis, in silico evaluation, and determination of the influence of the administration of these compounds in the in vivo diet-induced MetS model. 

## 2. Results 

### 2.1. Preparation of Compounds ***2***–***4***

**Scheme 1:**
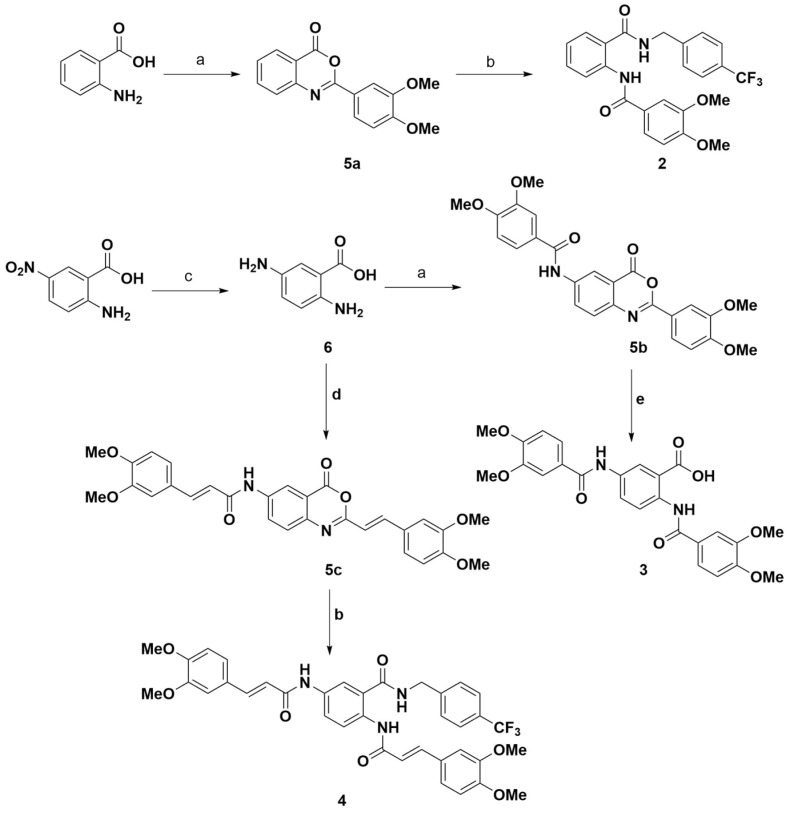
Preparation of target compounds **2**–**4. a.** 3,4-dimethoxybenzoyl chloride, DMF, room temperature, overnight; **b.** 4-(trifluoromethyl)benzylamine, DMF, room temperature, 3 h; **c.** Sodium hydrosulfite, 80 °C, NaOH 10%; **d.** 3,4-dimethoxybenzoyl chloride, DMF, room temperature, 24 h; **e**. NaOH 5%, room temperature, 1 h.

Scheme 1 illustrates the synthetic route to obtain compounds **2**–**4** based on our previous work [38]. 

Compound **2** was prepared from 4*H*-3,1-benzoxazin-4-one derivative **5a**, obtained from the reaction of anthranilic acid with 3,4-dimethoxybenzoic acid chloride (94% yield) and the subsequent opening of **5a** by treatment with 4-trifluoromethylbenzylamine to obtain compound **2** with a yield of 71%. A similar procedure was carried out to obtain compound **4** from compound **5c**, although slightly lower yields were obtained (71% for **5c** and 61% for **4**). The preparation of compound **3** was initially attempted from the treatment of 5-aminoanthranilic acid with two equivalents of 3,4-dimethoxybenzoic acid chloride, obtaining the targeted disubstituted compound with a small quantity of the mono-substituted derivative. Therefore, we decided to prepare **3** from compound **5b** (91% yield) and subsequent treatment with 5% sodium hydroxide solution to open the benzoxazinone ring, a strategy that gave better results in terms of yield (85%) and reaction workup.

### 2.2. In Silico Studies

Table 1 shows the results of the docking studies carried out on the projected targets. A more negative score value is associated with better binding. As expected, the decrease in molecular size usually leads to poorer binding ability, as seen for compound **2**, which displayed the lowest theoretical affinity of the four tested molecules, while compound **4** had the highest affinity. It is important to consider the concept of ligand efficiency (LE), which expresses the sensitivity of affinity to a variation in molecular size [54]. Table 1 displays LE in terms of the score divided by the number of heavy atoms. Based on these values, removing the 4-trifluoromethylbenzylamine fragment leads to more efficient ligands than the initial compound **1a**, suggesting that it is not critical for ligand binding. On the other hand, incorporating a vinyl moiety that delivers compound **4** has a detrimental effect on LE. Remarkably, compound **3** exhibited a similar LE to reference ligands. The in silico ADME/Tox profile was predicted using the pkCSM tool. Among the most relevant results, compound **3** would have the safer profile since it is not expected to be an hERG inhibitor, would have the highest tolerated dose, and would have higher metabolic stability than the other three compounds; however, it would possess the lowest intestinal absorption of the series (60% versus >80% of the other compounds). Overall, compound **3** rendered the best balance regarding predicted pharmacodynamic, pharmacokinetic, and toxicological properties.

**Figure 3:**
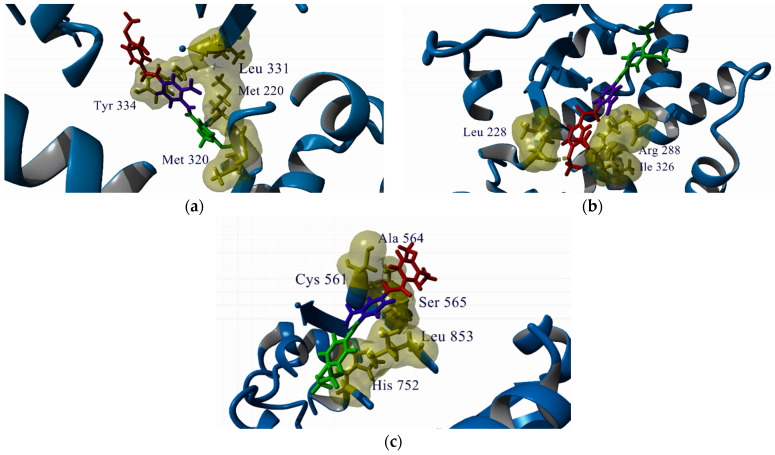
Predicted poses of compound **3** in the active site or ligand-binding domain (LBD) of the analyzed targets. (**a**) PPAR-α; (**b**) PPAR-γ; (**c**) HMG-CoA reductase. Key residues for ligand binding are labeled. The color code for compound **3** is the same as in Figure 2.

Figure 3 illustrates the predicted poses of compound **3** in the evaluated targets. The rest of the predicted poses are included as part of the Supplementary Materials. The 3,4-dimethoxybenzoyl groups occupy cavities described as necessary for the binding of the known ligands of these targets.

### 2.3. In Vivo and In Vitro Studies

**Figure 4:**
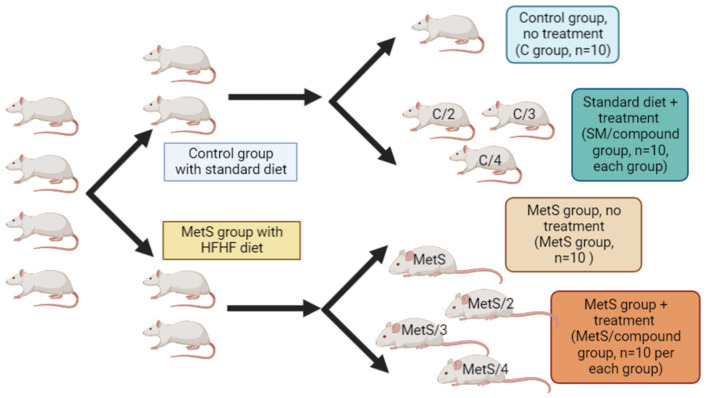
Experimental design for evaluation of compounds **2**–**4** in the diet-induced MetS model.

In the diet-induced model, MetS was generated through 12 weeks of the consumption of a high-fructose high-fat (HFHF) diet per the previous experience in our group [38,46]. The control group received a standard diet during the same period. After this induction phase, the animals of the standard diet were randomly allocated into two groups; one group (C/treated groups) would receive treatment with compounds **2**, **3**, or **4** for 14 days (10 mg/kg, p.o.), and the other group would receive no treatment (C groups). The same allocation was made for the diet-induced MetS group (the MetS group received no treatment, while MetS/treated groups received compound **2**, **3**, or **4**, 10 mg/kg, p.o. for 14 days), as depicted in Figure 4.

**Figure 5:**
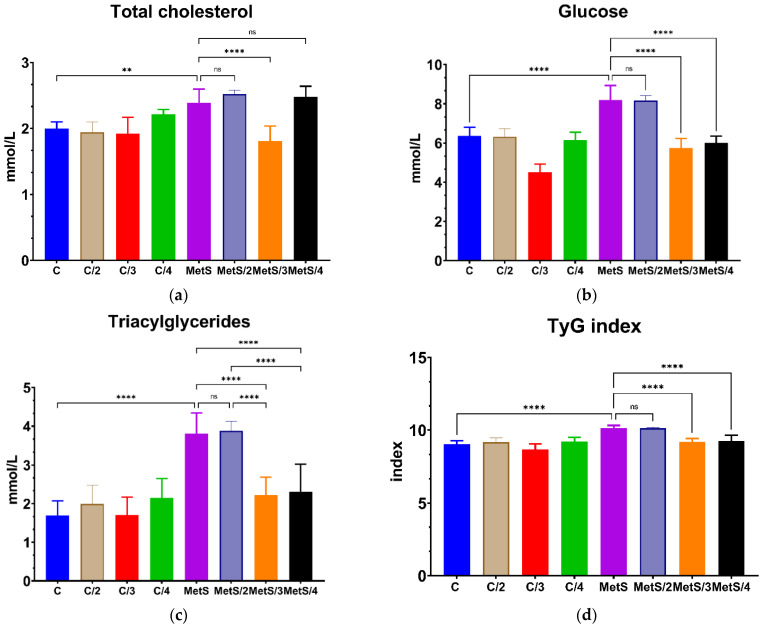
The effect after 14 days of administration of compounds **2**, **3** and **4** on metabolic parameters associated with MetS on the in vivo model. (**a**) Total cholesterol, (**b**) Glucose, (**c**) Triacylglycerides, (**d**) TyG index. Data are shown as means ± standard deviation (SD). The number of stars represents the level of significance differences between MetS groups and their MetS/treated groups; ns: not statistically significant difference (*p* > 0.05); ** *p* < 0.001; **** *p* < 0.0001.

The administration of compound **2** did not affect weight, glucose, cholesterol, or triglyceride levels; no statistical difference was observed compared with the control group (data not shown). The administration of compound **3** reduced body weight, glucose, cholesterol, and triglyceride levels, as seen in Figure 5. The weight and total cholesterol reduction were not significant in the group administered with compound **4**. Compound **3** even significantly lowered triglyceride levels, an effect not observed for compound **1** in our previous study. The triglycerides and glucose index was calculated according to the formula TyG = Ln(triglyceride (mg/dL)X glucose (mg/dL)/2 [55]; both compounds **3** and **4** induced a decrease in their value compared to the untreated group.

An initial screening of the in vitro inhibition of HMG-CoA reductase at 20 μM showed that compounds **3** and **4** inhibited the enzymatic activity (94% by compound **3** at 20 μM with an IC_50_ value of 8.89 ± 0.51 μM, and 46% by compound **4** at 20 μM). Then, we determined the in vitro antioxidant activity based on the determination of the Trolox equivalent antioxidant capacity (TEAC) using the cupric reducing antioxidant capacity (CUPRAC) assay; compound **4** exhibited higher activity (TEAC = 1.33 ± 0.04) than compound **3** (TEAC = 1.01 ± 0.06).

## 3. Discussion

Several examples of the optimization of an initial privileged scaffold for the discovery of multitarget drugs are extensively described in the literature [56,57,58,59,60,61]. In our case, we selected anthranilic acid as a privileged scaffold. According to our molecular docking-guided process, compound **2** exhibited the lowest theoretical affinity, which was reflected in the in vivo assay, being the only compound that did not demonstrate bioactivity at 10 mg/kg. This result suggests that the substitution in position 5 of the anthranilic acid core is required for in vivo activity. Remarkably, compound **3** simultaneously diminished total cholesterol, triglycerides, and glucose blood levels, showing an advantage over compounds **1** and **4**, which improved only two parameters.

**Figure 6:**
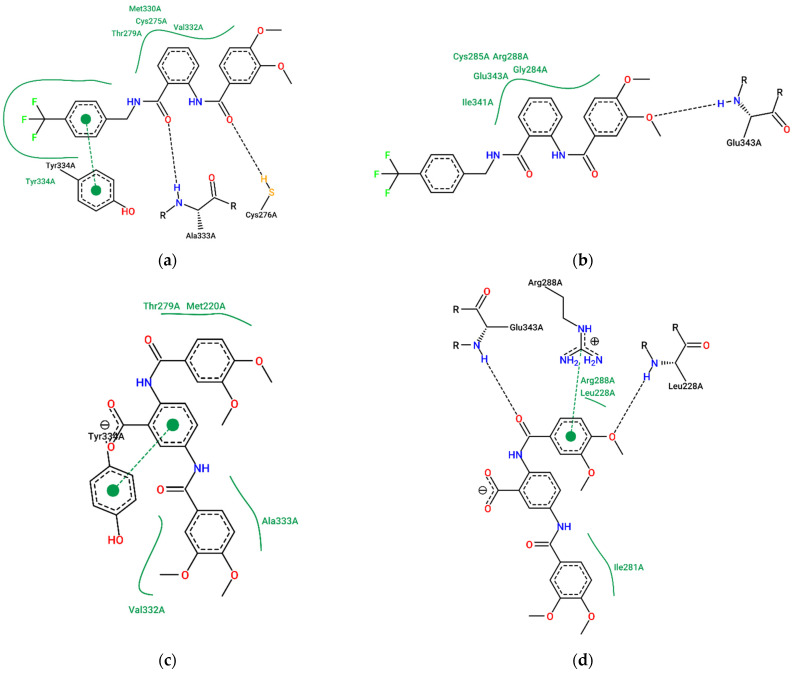
Comparison of the predicted complexes of compound **1** with (**a**) PPAR-α and (**b**) PPAR-γ; complexes of compound **3** with (**c**) PPAR-α and (**d**) PPAR-γ. Diagrams generated using the ProteinPlus portal [62].

To understand the structural factors that could explain these differences in in vivo activity, we compared the binding modes of compounds **1**–**4**. Figure 6 illustrates the predicted poses of compounds **2** and **3** within the LBD of PPAR-α and PPAR-γ.

It has been described that the LBD of PPAR receptors is Y-shaped, meaning that it has three major cavities [63,64,65,66]. Compound **2** can only interact with one of these cavities, the cavity close to Cys 276 in the case of PPAR-α and Cys 285 in PPAR-γ. Meanwhile, compounds **1**, **3**, and **4** interact with at least two of them: additionally to the cavity occupied by compound **2**, the additional aromatic ring occupies the cavity close to Met 220 in PPAR-α and Arg 288 in PPAR-γ. Thus, this is a plausible explanation for the lack of activity of compound **2**. Interestingly, the 4-trifluoromethylbenzylamide moiety of compounds **1** and **4** is not required to occupy these cavities, suggesting that it can be removed, and the bioactivity would be maintained as demonstrated by the in vivo experimentation. The dose of 10 mg/kg administrated during this study showed effects on glucose and triglyceride levels similar to those caused by comparable doses of other PPAR agonists in animal models, such as glitazones such as pioglitazone and rosiglitazone (3–10 mg/kg) [67,68] or fibrates such as fenofibrate and bezafibrate (20–50 mg/kg) [69,70]. 

**Figure 7:**
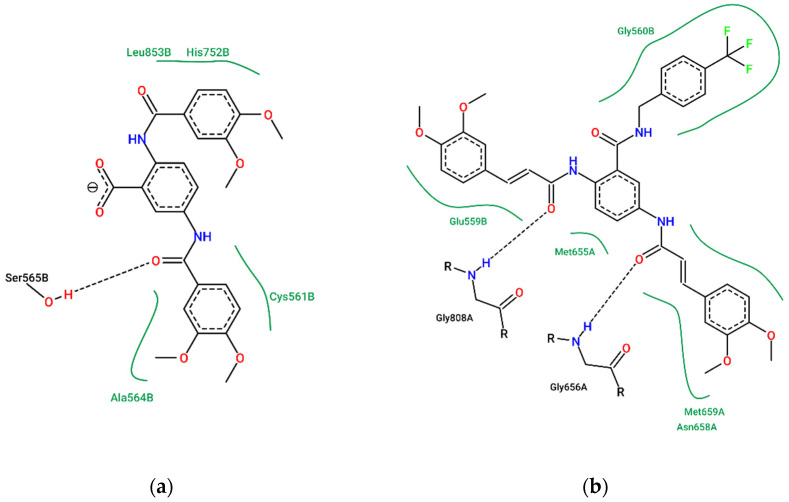
Comparison of the predicted complexes of (**a**) compound **3** and (**b**) compound **4** with HMG-CoA reductase. Diagrams generated using the ProteinPlus portal [62].

Compound **3** exhibited the highest effect on total cholesterol levels. Figure 7 depicts the predicted binding mode of compounds **3** and **4** within the active site of HMG-CoA reductase. One of the 3,4-dimethoxyphenyl moieties of compounds **1** and **3** occupies the cavity where the mevalonate binds to HMG-CoA reductase (close to Ser 684 and His 752), while the other 3,4-dimethoxyphenyl moiety occupies the same site as the pyrrole ring of atorvastatin (close to Ser 565 and Cys 561). On the other hand, due to its higher molecular volume, compound **4** cannot bind to the site where mevalonate fits; this could explain the lack of effect of compound **4** in total cholesterol levels.

In our hands, using a commercial kit to determine the inhibitory activity on HMG-CoA reductase, compound **3** had an IC_50_ of 8.89 μM. Cao et al. informed that the IC_50_ values of statins on HMG-CoA reductase activity are reported in the range of several nanomolar to several micromolar levels [71,72]. Also, Mendieta et al. reported IC_50_ values for α-asarone and simvastatin of 5.86 μM and 6.11μM, respectively, using the same commercial kit [73]. Therefore, compound **3** exhibits similar in vitro HMG-CoA inhibition and in vivo antihypercholesterolemic properties to other hypolipidemic agents. The antihypercholesterolemic activity of both α-asarone (80 mg/kg) [74] and simvastatin (10 mg/kg) [75,76] has been demonstrated in animal models of hypertension and obesity, with results comparable to those obtained with compound **3**.

Previously, De las Heras et al. reported that treatment with rosuvastatin reduced plasma cholesterol and triacylglyceride levels in animals fed with a hypercaloric diet, enhancing PPAR-γ expression. PPAR-α agonists such as gemfibrozil, clofibrate, fenofibrate, and fenofibric acid have also been shown to have a similar effect. These compounds substantially decrease plasma TG levels and increase HDL levels. It is appropriate to think that the effect observed in compounds **3** and **4** results from an orchestrated and balanced activation between different PPAR subtypes and, in the case of compound **3**, from its HMG-CoA inhibition properties.

The triglyceride glucose (TyG) index is a parameter obtained from fasting triglyceride (TG) and plasma glucose levels. It has been proposed as a surrogate marker of MetS and insulin resistance due to its sensitivity, precision, and specificity compared to the HOMA-IR index and the gold standard euglycemic hyperinsulinemic clamp test [77,78,79]. It has performed better in forecasting the development of diabetes mellitus type II (DMII) than the values of fasting glucose and triglycerides alone [80]. Recently, it has been suggested as a parameter to evaluate the early effects of dietary intervention or antioxidant treatment [81,82]. As seen in Figure 5d, the HFHF diet significantly increases the TyG index value compared to the group with the standard diet, as expected. Due to their effects on glucose and triglyceride levels, both compounds **3** and **4** lowered the TyG index, suggesting they would impact insulin resistance and other factors related to the onset and development of MetS.

An oxidant/antioxidant disparity may influence the development of MetS, since it has been observed that patients suffering from MetS display higher levels of oxidative damage markers along with the reduced activity of antioxidant enzymes [83,84]. Also, there is evidence of the positive effect of antioxidant administration in hypertension and MetS [85,86,87]. Therefore, it would be desirable that a multitarget drug designed for managing MetS would have antioxidant properties, as it was reported for multitarget drugs focused on Alzheimer’s disease [88]. For the estimation of the total antioxidant capacity (TAC), we used a commercial kit based on the cupric reducing antioxidant capacity (CUPRAC) spectrophotometric method. Our results suggest that both compounds **3** and **4** have antioxidant activity similar to ferulic acid (TEAC = 1.20) [89] but lower than more potent antioxidants quercetin or epicatechin gallate (TEAC values above 3.0). Further investigation is needed to demonstrate if the antioxidant activity of these compounds is related to their positive effects in MetS.

## 4. Materials and Methods

### 4.1. In Silico Studies

#### Docking Studies

The molecular structures were built using ACDLabs (Advanced Chemistry Development, Inc., Toronto, ON, Canada), optimized using MMFF//HF 6-31G* in Spartan 10 for Windows (Wavefunction, Inc., Irvine, CA, USA) and saved as mol2format. Then, these files were exported to Molegro Virtual Docker 6.0.1 (Qiagen Bioinformatics, Aarhus, Denmark). The molecular docking studies were carried out in the following targets using the accession codes shown in parenthesis: HMG CoA reductase (PDB ID: 1HWK), PPAR-α (PDB ID: 1I7G), and PPAR-γ (PDB ID: 1I7I). A previously reported procedure was used [26,90]. Briefly, all the co-crystallized ligands and water molecules were deleted from the structures. The ligand-binding domain (LBD) of the nuclear receptors or the active sites of each enzyme were selected as the searching sites under a 15 Å radius sphere. The MolDock Optimizer algorithm was set to 5000 maximum iterations with a simplex evolution population of 5000 and 50 runs for each ligand. Rerank Score was calculated as the criteria for estimating the theoretical binding affinity. Better binding is associated with more negative scores. The co-crystallized ligands were also redocked to their respective receptors to assess the efficacy of this procedure. In all the docking procedures, the RMSD of the co-crystallized ligand pose compared with the original structure was lower than 2.0 Å. The pkCSM online platform [91] was used to predict the in silico ADME/Tox profile. (http://biosig.unimelb.edu.au/pkcsm/, accessed on 10 June 2022). 

### 4.2. Chemistry

All initial materials for the synthesis of compounds **2**–**4** were purchased from Sigma-Aldrich (St. Louis, MO, USA). Monitoring of the synthetic transformations was carried out using TLC on silica gel 60 on aluminum foils, also from Sigma-Aldrich. Infrared (IR) spectra were acquired in a Perkin Elmer FTIR-670 Plus spectrophotometer in ATR. ^1^H and ^13^C NMR spectra were recorded using a JEOL ECA-500 JEOL spectrometer (B_0_ = 11.75 T) in a DMSO-d_6_ solution. ^1^H NMR spectra and ^13^C NMR were recorded at 500.1599 MHz and 125.7653 MHz, respectively. The FAB-MS analyses were performed on a JEOL Sx102 mass spectrometer. The initial precursor 2,5-diaminobenzoic acid was prepared as previously reported [38].

#### 4.2.1. General Procedure for the Preparation of Benzoxazinone Derivatives **5a**–**c**

**Figure 8:**
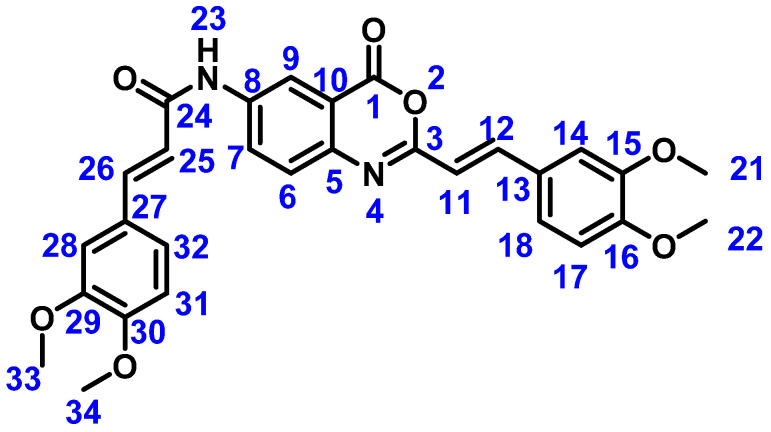
Structure of novel compound **5c** with atom numbering used for NMR characterization.

Benzoxazinone derivatives were prepared as previously reported. Briefly, to a round bottom flask with magnetic agitation and ice bath, 100 mL of pyridine was added. After that, 26.0 mmol of 3,4-dimethoxybenzoyl chloride or 3,4-dimethoxycinnamoyl chloride was added and stirred until the formation of a yellowish solution. After 10 min, freshly prepared 2,5-diaminoanthranilic acid (1.3 g, 8.7 mmol) was added, and the resulting suspension was stirred for an additional 2 h at 5 °C. Once the reaction was finalized, 250 mL of water was added to the reaction mixture, and a precipitate was immediately formed, which was then filtered, washed with ethanol, and dried to afford the desired product as a yellowish solid. 

(*E*)-3-(3,4-dimethoxyphenyl)-*N*-(2-((*E*)-3,4-dimethoxystyryl)-4-oxo-4*H*-benzo[*d*][1,3]oxazin-6-yl)acrylamide (**5c**). IR (ATR, cm^−1^): 3343 (N-H), 2889 (C=C), 1759 (C=O), 1684 (C=O, amide), 1586 (C=C, aromatic), 1134 (O-CH_3_). ^1^H NMR (DMSO-d_6_, 500 MHz) δ: 10.55 (1H, s, H23), 8.56 (1H, d, *J* = 2.4 Hz, H9), 7.99 (1H, dd, *J* = 2.4, 8.8 Hz, H7), 7.62 (1H, d, *J* = 16.0 Hz, H12), 7.54 (1H, d, *J* = 8.8 Hz, H6), 7.54 (1H, d, *J* = 15.5 Hz, H26), 7.40 (1H, d, *J* = 1.7 Hz, H14), 7.27 (1H, dd, *J* = 1.7, 8.4 Hz, H18), 7.19 (1H, d, *J* = 1.7 Hz, H28), 7.17 (1H, dd, *J* = 1.7, 8.4 Hz, H32), 6.98 (1H, d, *J* = 8.4 Hz, H31), 6.95 (1H, d, *J* = 8.4 Hz, H17), 6.87 (1H, d, *J* = 16.0 Hz, H11), 6.65 (1H, d, *J* = 15.5 Hz, H25), 3.80 (3H, s, H21), 3.79 (3H, s, H33), 3.76 (6H, s, H22, H34). ^13^C {1H} NMR (DMSO-*d_6_*, 125 MHz) δ: 164.72 (C24), 159.49 (C1), 156.50 (C3), 151.33 (C16), 151.10 (C30), 149.55 (C15), 149.43 (C29), 142,28 (C5), 141.67 (C26), 141.10 (C12), 139.59 (C8), 128.04 (C7, C13), 127.84 (C6), 127.76 (C27), 123.28 (C18), 122.57 (C32), 119.67 (C25), 117.57 (C10), 117.23 (C11), 116.87 (C9), 112.22 (C31), 112.08 (C17), 110.69 (C14), 110.51 (C28), 56.13 (33), 56.07 (34), 55.92 (21), 55.45(22). See Figure 8 for atom numbering. MS (FAB, *m*/*z*): 515 (M^+^ + 1, 100%). Elemental analysis: experimental C, 66.17%: H, 5.18%; N, 5.12%; calculated C, 67.70%; H, 5.09%; N, 5.44%.

#### 4.2.2. 2,5-Bis(3,4-dimethoxybenzamido)benzoic Acid (Compound **3**)

**Figure 9:**
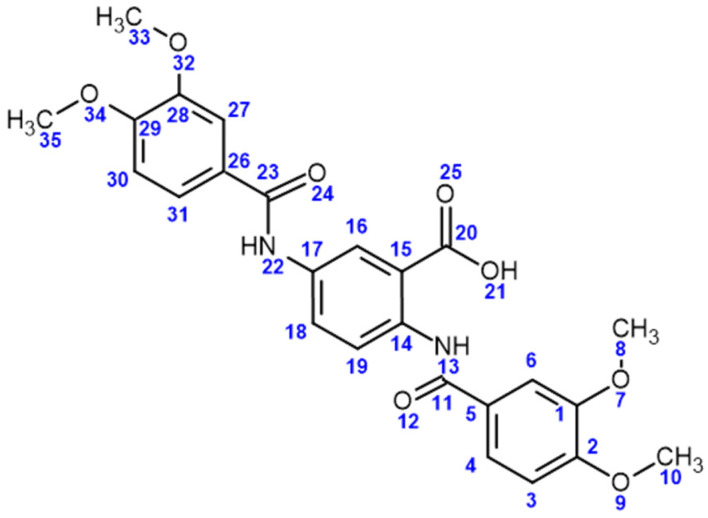
Structure of compound **3** with atom numbering used for NMR characterization.

*Method A*: In a round-bottom flask, 1.3225 g of 3,4-dimethoxybenzoyl chloride (6.592 mmol) was added to 10 mL of pyridine at room temperature. Next, 0.5 g of 2,5-diaminobenzoic acid (3.2882 mmol) was added to the solution, obtaining a beige -colored suspension. The reaction mixture was stirred for 24 h at room temperature. Afterward, once the reaction was completed, the crude was transferred to a beaker with 20 mL of water and was treated with chloride acid until it reached pH 2, rendering a white precipitate that was filtered and dried under reduced pressure to render the desired product as a white solid in a 35% yield. *Method B*: Given the low yield obtained from the previous synthesis route for compound **3**, an alternative synthesis route was proposed from the basic hydrolysis of the benzoxazinone ring of compound **5a**, prepared as previously reported [38]. In a round-bottom flask with 3 mL of DMF, 0.2 g of compound **5a** was added, obtaining a milky white suspension. Next, 10 mL of a 5% NaOH solution was slowly added, giving place to a white suspension. The reaction mixture was stirred for 2 h at room temperature, followed by the treatment of the crude with diluted HCl solution until it reached pH 2. The acid treatment of the crude resulted in the formation of a white precipitate that was filtered and dried under reduced pressure to give the desired product as a white solid in an 85% yield. m.p 289–292 °C; IR (ATR, cm^−1^): 3377 (N-H); 3125.14 (C-H sp^2^), 2932.72 (C-H sp^3^), 1654.05 (C=O amide), 1601.18 (C=C aromatic), 1189.74 (C-O); ^1^H NMR (DMSO-*d*_6_, 500 MHz) *δ*: 12.23 (1H, s, H13), 10.18 (1H, s, H22), 8.62 (1H, d, *J* = 8.8 Hz, H19), 8.45 (1H, d, *J* = 2.5 Hz, H16), 7.94 (1H, dd, *J* = 8.8, 2.5 Hz, H18), 7.58 (1H, dd, *J* = 8.5, 1.8 Hz, H4), 7.51 (1H, d, *J* = 1.8 Hz, H6) 7.50 (1H, dd, *J* = 8.5, 1.8 Hz, H31), 7.45 (1H, d, *J* = 1.8 Hz, H27), 7.05 (1H, d, *J* = 8.5 Hz, H3), 7.01 (1H, d, *J* = 8.5 Hz, H30), 3.80 (6H, s, H10, H35), 3.79 (3H, s, H8), 3.76 (3H, s, H32). ^13^C{^1^H} NMR (DMSO-*d*_6_, 125 MHz) *δ*: 170.60 C20), 165.49 (C23), 164.59 (C11), 152.39 (C2), 152.14 (C29), 149.10 (C1), 148.73 (C28), 137.60 (C14), 134.38 (C17), 127.29 (C5), 127.04 (C26), 126.57 (C18), 123.59 (C16), 121.62 (C4), 120.69 (C31), 120.30 (C19), 117.53 (C15), 111.70 (C3), 111.31 (C6, C30), 110.63 (C27), 56.18 (C10), 56.12 (C35), 56.09 (C8), 55.95 (C32). See Figure 9 for atom numbering. MS (FAB, *m*/*z*): 481 (M^+^ + 1, 100%). Elemental analysis: experimental C, 61.18%: H, 5.12%; N, 5.47%; calculated C, 62.50%; H, 5.04%; N, 5.83%.

#### 4.2.3. (2*E*,2′*E*)-*N,N*′-(2-((4-(Trifluoromethyl)benzyl)carbamoyl)-1,4-phenylene)bis(3-(3,4-dimethoxyphenyl)acrylamide) (Compound **4**)

**Figure 10:**
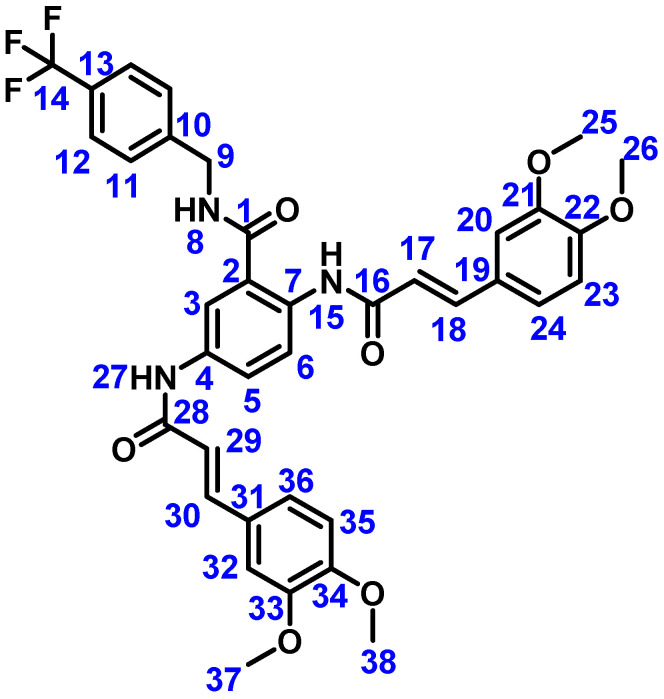
Structure of compound **4** with atom numbering used for NMR characterization.

In a round-bottom flask equipped with magnetic agitation, 0.25 g (0.5 mmol) of compound **5c** was dissolved in 15 mL of DMF, and 0.7 mmol of 4-trifluorobenzylamine was added. The reaction mixture was stirred at room temperature for 24 h. During the reaction, the amber solution turns into a yellowish suspension after completion. After the reaction was completed, 30 mL of water was added to render a white precipitate that was washed with acetone and subsequently with methanol. Yield: 61%, m.p. >250 °C. IR (ATR, cm^−1^): 3377 (N-H); 3125.14 (C-H), 2932.72 (C-H), 1654.05 (C=O amide), 1601.18 (C=C aromatic), 1189.74 (C-O); ^1^H NMR (DMSO*-d_6_*, 500 MHz) δ: 10.66 (1H, s, H15), 10.26, (1H, s, H27), 9.33 (1H, t, *J* = 5.5 Hz, H8), 8.27 (1H, d, *J* = 8.9 Hz, H6), 8.09 (1H, *J* < 2 Hz, H3), 7.71 (1H, d, *J* = 8.9 Hz, H5), 7.67 (2H, d, *J* = 8.0 Hz, H12) 7.54 (2H, d, *J* = 8.1 Hz, H11), 7.51 (1H, d, *J* = 15.0 Hz, H30), 7.48 (1H, d, *J* = 15.2 Hz, H18), 7.31 (1H, *J* < 2 Hz, H20), 7.18 (1H, *J* < 2 Hz, H32), 7.17 (1H, d, *J* = 8.4 Hz, H24), 7.15 (1H, d, *J* = 8.3 Hz, H36), 6.98 (1H, d, *J* = 8.3 Hz, H35), 6.95 (1H, d, *J* = 8.4 Hz, H23), 6.73 (1H, d, *J* = 15.2 Hz, H17), 6.68 (1H, d, *J* = 15.0 Hz, H29), 4.54 (2H, d, *J* = 5.5 Hz, H9), 3.79 (3H, s, H37), 3.79 (3H, s, H38), 3.76 (3H, s, H25), 3.75 (3H, s, H26). ^13^C {1H} NMR (DMSO-*d_6_*, 125 MHz) δ: 168.80 (C1), 164.41 (C28), 164. 37 (C16), 151.03 (C33), 150.98 (C21), 149.47 (C22), 149.44 (C34), 144.58 (C10), 141.61 (C18), 141.00 (C30), 135.22 (C7), 133.99 (C4), 128.47 (C11), 127.92 (C19), 127.77 (C13), 127.88 (C31), 125.78 (C12), 124.26 (C2) 123.76 (C14), 123.05 (C24), 122.93 (C6), 122.88 (C5), 122.35 (C36), 120.33 (C17), 120.06 (C29), 119.46 (C3), 112.27 (C35), 112.05 (C23), 110.65 (C20), 110.53 (C32), 56.09 (C37, C38), 56.06 (C26), 55.93 (C25), 42.87 (C9). 19F {1H} NMR (DMSO-*d_6_*, 470 MHz) δ: 4.32 (F). See Figure 10 for atom numbering. MS (FAB, *m*/*z*): 690 (M^+^ + 1, 100%). Elemental analysis: experimental C, 62.94%: H, 5.11%; N, 5.60%; calculated C, 64.44%; H, 4.97%; N, 6.09%.

### 4.3. In Vivo Evaluation in Metabolic Syndrome

#### 4.3.1. Animals

For the in vivo experimentation, eighty Sprague–Dawley male rats weighing 250 ± 25 g were housed in acrylic boxes and treated following the recommendations and requirements of the NOM-062-ZOO-1999 (Official Mexican Standard for the Production, Care, and Use of Laboratory Animals). They were kept in a clear-air room maintained on an artificial 12 h light/dark cycle and were given ad libitum access to food and water. The protocol was approved by the Institutional Subcommittee for the Care and Use of Laboratory Animals (SICUAL) with the registration number FMM/SICUAL/006/2017 (date of approval, 30 August 2017).

#### 4.3.2. MetS Induction and Treatment with the Tested Compounds

The animals were randomly allocated to a group (control groups) that was fed on a regular Chow commercial diet (Purina-Rodent Laboratory Chow-5001 3.310 kcal/g) or to a group (MetS groups) that was supplied with a high-fructose and high-fat diet (4.161 kcal/g) for the generation of MetS. From our own experience, MetS is established after twelve weeks with this diet [46]. The weight of each animal was recorded at the beginning of the study and every week after the completion of the induction period. In weeks nine and twelve, the glucose, total cholesterol, and triglyceride levels were monitored in fasting conditions of 12 h, using an Accutrend plus monitor (Roche). Following the MetS induction phase, the control and MetS groups were rearranged into the following groups: (1) control (CM, regular diet + no treatment n = 10); (2) MetS (HFHF diet + no treatment, n = 10); (3) control + compound **2** (C/2 regular diet + treatment with **2**, n = 10); (4) MetS + compound **2** (MetS/2, HFHF diet + treatment with **2**, n = 10); (5) control + compound **3** (C/3, regular diet + treatment with **1c**, n = 10); (6) MetS + compound **3** (MetS/3, HFHF diet + treatment with **3**, n = 10); (7) control + compound **4** (C/4, regular diet + treatment with **4**, n = 10); (8) MetS + compound **4** (MetS/4, HFHF diet + treatment with **4**, n = 10). The treatment stage lasted 14 days, and the animals retained the same diet they had during the induction to exclude the effect of diet change. All the animals received 100 μL of a 1% Kolliphor EL mixture in water with or without the tested compound (10 mg/Kg) via nasogastric. Animals were sacrificed by decapitation. The blood was collected and centrifuged at 1372× *g* for 15 min at 4 °C. The serum was gathered and stored at −80 °C until the determination of metabolic parameters.

#### 4.3.3. Triacylglycerides, Cholesterol, and Glucose Analysis

The animals’ serum was defrosted, and triacylglyceride, cholesterol, and glucose levels were determined on an Architect c8000 system (Abbott, Wiesbaden, Germany). Statistical analysis was performed using one-way ANOVA, followed by Dunnett’s multiple comparisons tests employing GraphPad Prism version 9.4.1 (GraphPad Software, La Jolla, CA, USA). Differences were considered significant when *p* ≤ 0.05.

### 4.4. In Vitro Evaluation of HMG-CoA Reductase Inhibition and Antioxidant Activity

The HMG-CoA Reductase Assay Kit from Sigma-Aldrich (catalog number CS1090, St. Louis, MO, USA) was used to determine the inhibitory properties of compounds **3** and **4** over enzyme activity, using the instructions provided by the fabricant. An initial screening was performed at a final concentration of 20 μM, resulting in a 94% inhibition of enzyme activity by compound **3** and 46% by compound **4**. For IC_50_ determination of compound **3**, final concentrations of 2.5, 5.0, 10.0, and 20 μM were employed by triplicate. The Total Antioxidant Capacity Assay Kit from Sigma-Aldrich (catalog number MAK187, St. Louis, MO, USA) was used for the determination of in vitro antioxidant activity following the manufacturer’s instructions by triplicate.

## 5. Conclusions

**Figure 11:**
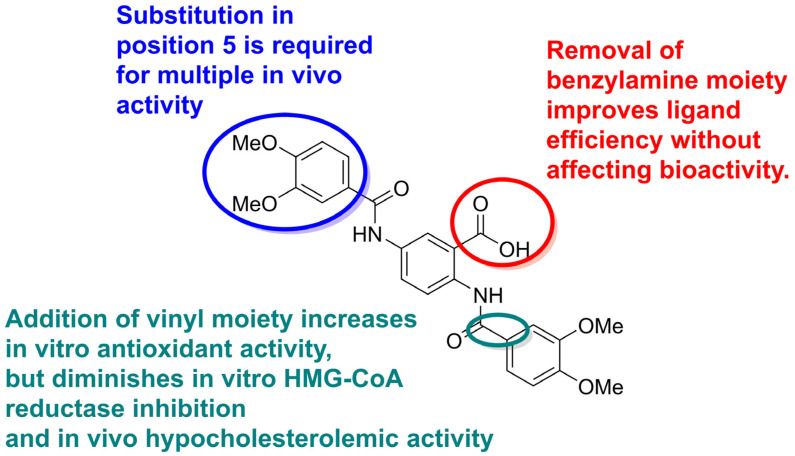
Structural features that modify biological activity on 5-aminoanthranilic acid derivatives.

In conclusion, we designed compound **3**, an anthranilic acid derivative, as a hit in developing new multitarget drugs for the management of MetS. The most relevant findings are summarized in Figure 11. We found that substitution in position 5 is needed for in vivo activity over at least two parameters related to MetS. Removing substituents in the carboxylic group leads to higher in silico ligand efficiency and improves in vivo polypharmacology activity. On the other hand, incorporating a vinyl group besides the 3,4-dimethoxyphenyl group increases antioxidant properties but diminishes the inhibition of HMG-CoA reductase activity, which is reflected in the loss of in vivo activity over cholesterol levels. The accumulated evidence of this work suggests that the underlying mechanisms related to the biological effects of these new compounds are related to numerous pleiotropic actions, including inflammatory response, lipid metabolism, glucose metabolism, and the regulation of oxidative stress. According to this idea, the observed in vivo effects result from an orchestrated and balanced activation between different PPAR subtypes and HMG-CoA inhibitory properties.

## Figures and Tables

**Table 1:** Results of molecular docking studies.

Compound	PPAR-α	LE	PPAR-γ	LE	HMG-CoA Reductase	LE
**1**	−151.6	−3.3	−165.2	−3.6	−156.2	−3.4
**2**	−119.8	−3.6	−109.8	−3.3	−135.2	−4.1
**3**	−121.8	−3.5	−125.4	−3.6	−171.4	−4.8
**4**	−132.7	−2.6	−144.5	−2.9	−211.5	−4.2
**Reference ligands** ^1^	−112.4	−4.0	−109.0	−3.9	−156.1	−3.8

^1^ Reference ligands: Terglitazaar for PPAR-α, rosiglitazone for PPAR-γ, atorvastatin for HMG-CoA reductase.

## Data Availability

Data is contained within the article and Supplementary Materials.

## Supplementary Materials

The following supporting information can be downloaded at: https://www.mdpi.com/article/10.3390/ph15121461/s1: predicted poses of compounds **2**–**4** to the studied targets, weight variations during administration of compounds **3** and **4**, NMR spectra of compounds **3** and **4**.
