# Supplementary material for: In Combo Studies for the Optimization of 5-Aminoanthranilic Acid Derivatives as Potential Multitarget Drugs for the Management of Metabolic Syndrome
Source: Pharmaceuticals (Basel). 2022 Nov 25;15(12):1461. doi: 10.3390/ph15121461 (PMC9784827; doi:10.3390/ph15121461)
Supplement: Supplementary file 1 [file pharmaceuticals-15-01461-s001.zip › pharmaceuticals-2036712-supplementary-1.pdf]

Supplementary material.

1. Predicted poses for compounds 2, 3 and 4 con the analyzed targets

PPAR-alpha

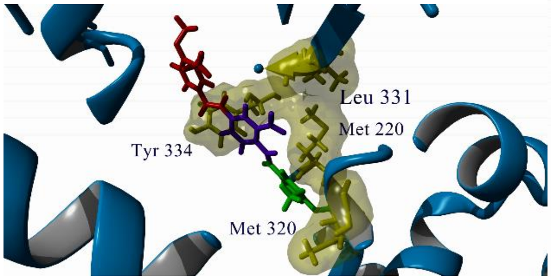

Compound **3**

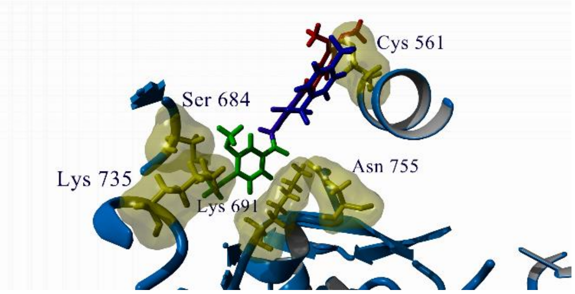

Compound **4**

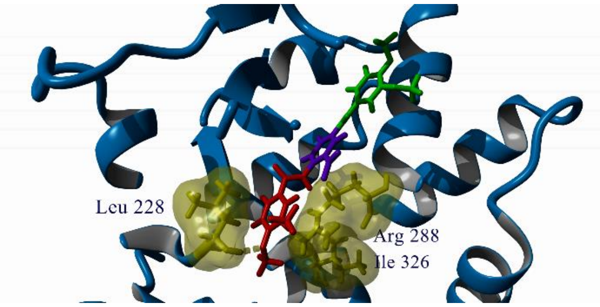

Compound **3**

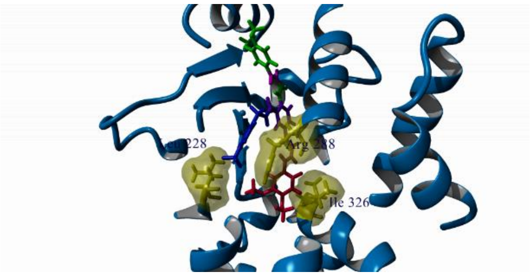

Compound **4**

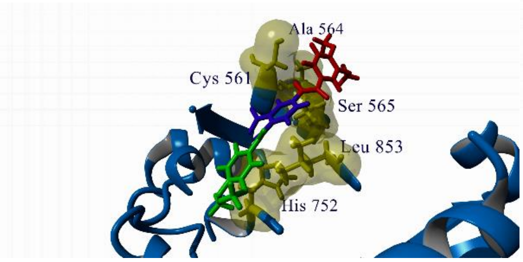

Compound **3**

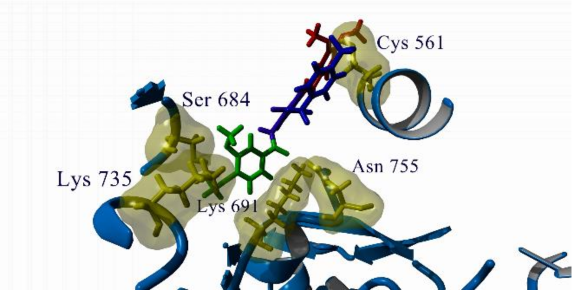

Compound **4**

2. Weight variation during MetS induction and treatment with compounds **3** and **4** periods.

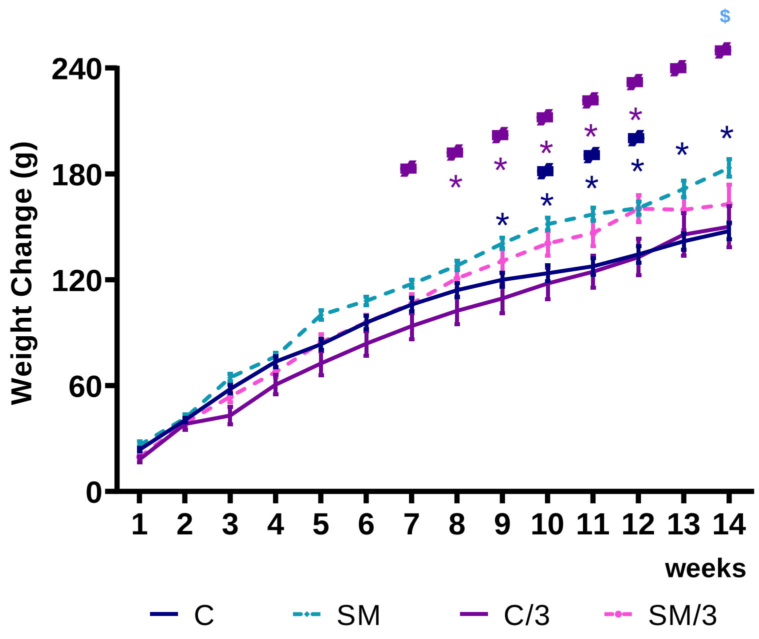

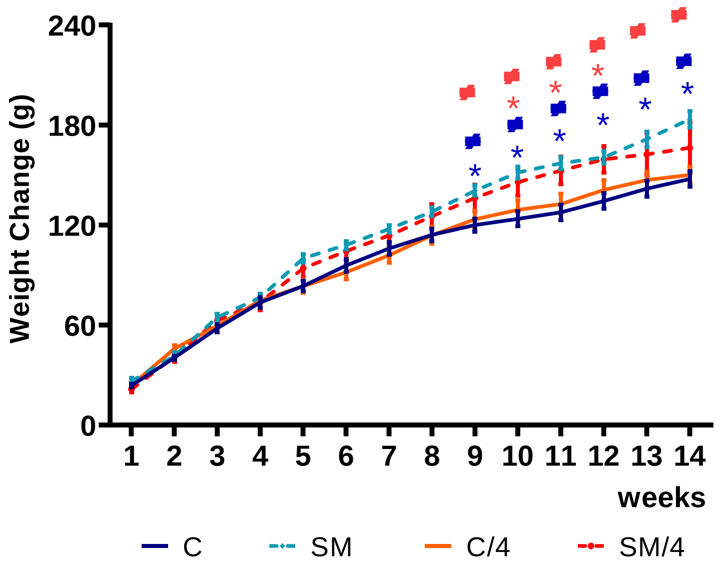

3. NMR spectra for compounds **3** and **4**

3.1 Compound **4**

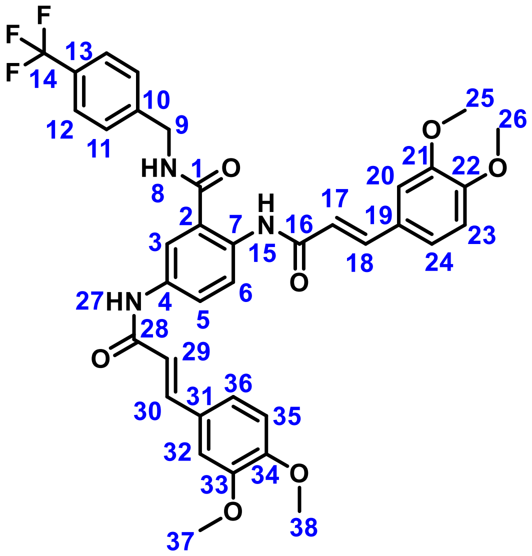

^{1}H NMR (DMSO-d$_6$, 500 MHz) δ: 10.66 (1H, s, **H15**), 10.26, (1H, s, **H27**), 9.33 (1H, t, J = 5.5 Hz, **H8**), 8.27 (1H, d, J = 8.9 Hz, **H6**), 8.09 (1H, $J_{H3,H5}$<2 Hz, **H3**), 7.71 (1H, d, J = 8.9 Hz, **H5**), 7.67 (2H, d, J = 8.0 Hz, **H12**) 7.54 (2H, d, J = 8.1 Hz, **H11**), 7.51 (1H, d, J = 15.0 Hz, **H30**), 7.48 (1H, d, J = 15.2 Hz, **H18**), 7.31 (1H, $J_{H3,H5}$<2 Hz, **H20**), 7.18 (1H, $J_{H3,H5}$<2 Hz, **H32**), 7.17 (1H, d, J = 8.4 Hz, **H24**), 7.15 (1H, d, J = 8.3 Hz, **H36**), 6.98 (1H, d, J = 8.3 Hz, **H35**), 6.95 (1H, d, J = 8.4 Hz, **H23**), 6.73 (1H, d, J = 15.2 Hz, **H17**), 6.68 (1H, d, J = 15.0 Hz, **H29**), 4.54 (2H, d, J = 5.5 Hz, **H9**), 3.79 (3H, s, **H37**), 3.79 (3H, s, **H38**), 3.76 (3H, s, **H25**), 3.75 (3H, s, **H26**).

^{13}C {^{1}H} NMR (DMSO-d$_6$, 125 MHz) δ: 168.80 (**C1**), 164.41 (**C28**), 164. 37 (**C16**), 151.03 (**C33**), 150.98 (**C21**), 149.47 (**C22**), 149.44 (**C34**), 144.58 (**C10**), 141.61 (**C18**), 141.00 (**C30**), 135.22 (**C7**), 133.99 (**C4**), 128.47 (**C11**), 127.92 (**C19**), 127.77 (**C13**), 127.88 (**C31**), 125.78 (**C12**), 124.26 (**C2**) 123.76 (**C14**), 123.05 (**C24**), 122.93 (**C6**), 122.88 (**C5**), 122.35 (**C36**), 120.33 (**C17**), 120.06 (**C29**), 119.46 (**C3**), 112.27 (**C35**), 112.05 (**C23**), 110.65 (**C20**), 110.53 (**C32**), 56.09 (**C37, C38**), 56.06 (**C26**), 55.93 (**C25**), 42.87 (**C9**).

^{19}F {^{1}H} NMR (DMSO-d$_6$, 470 MHz) δ: 4.32 (**F**).

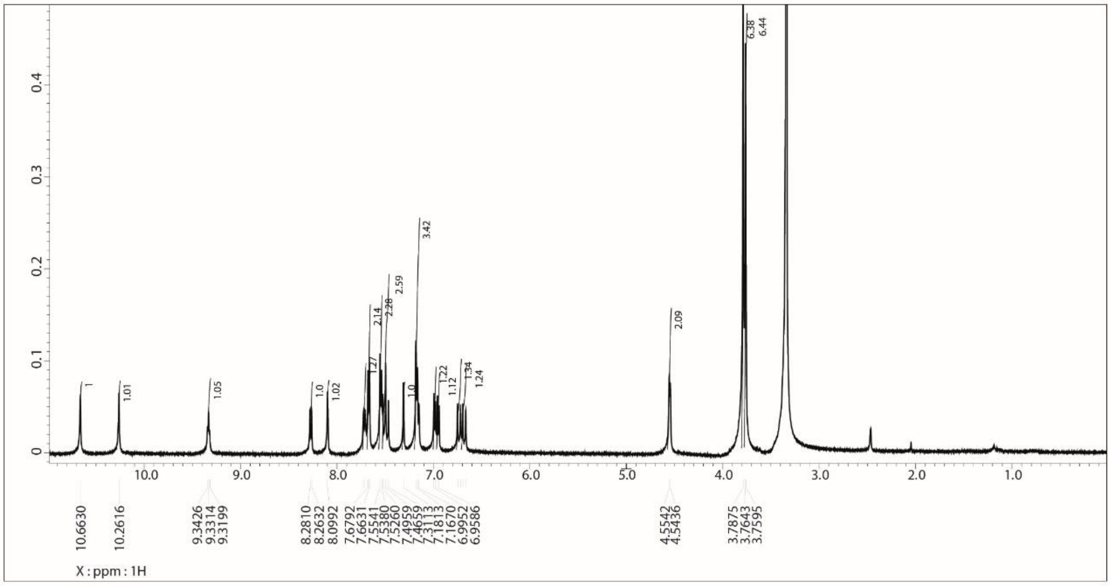
X : ppm : 1H

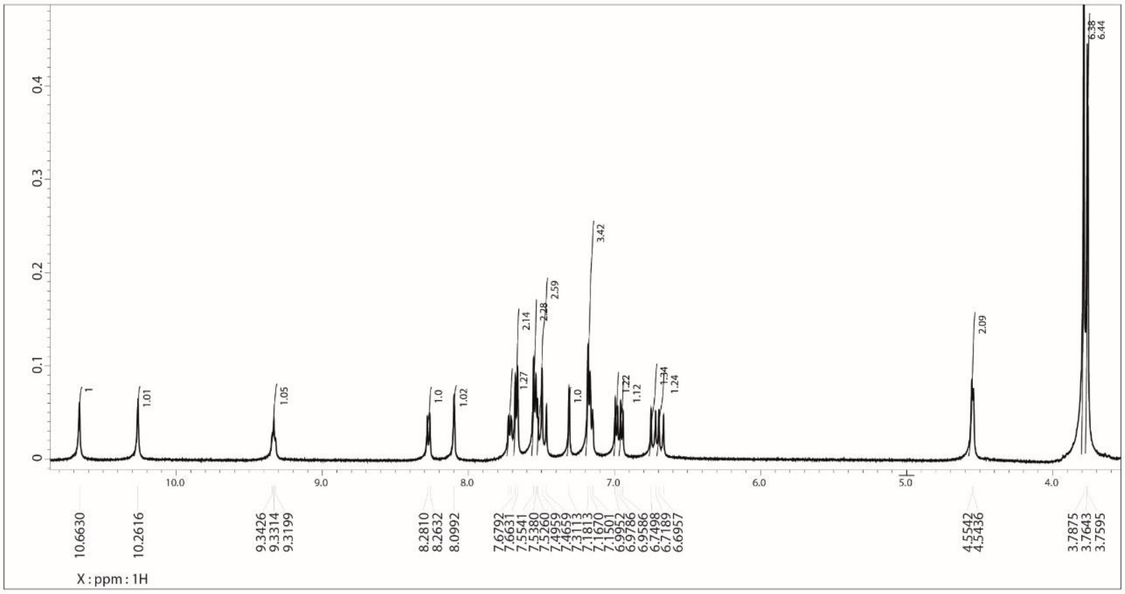
X : ppm : 1H

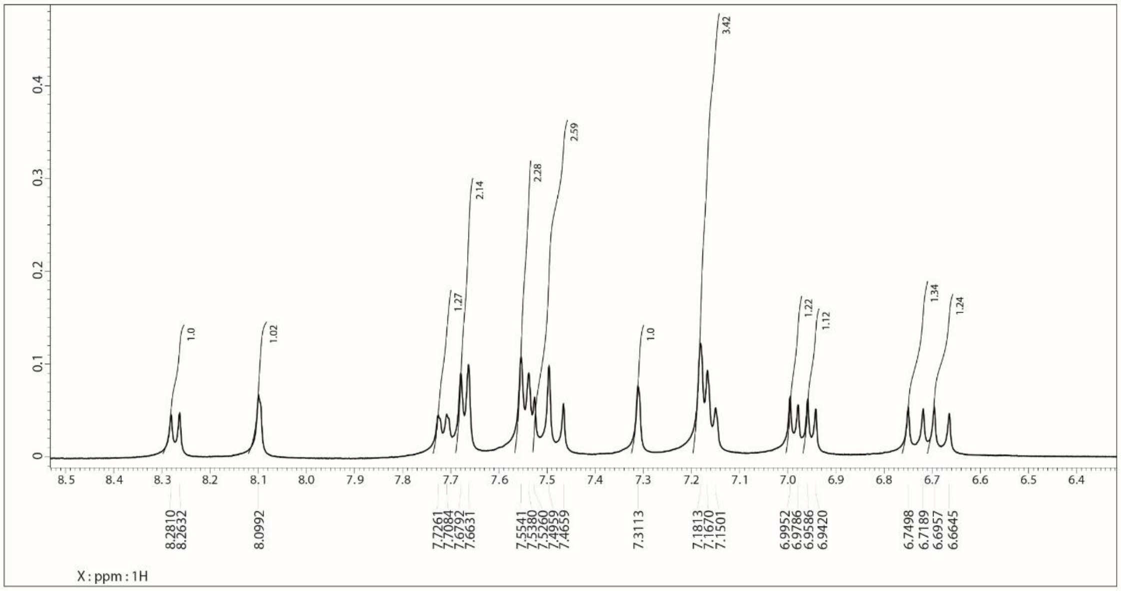

1.0
1.02
1.27
2.14
2.28
2.59
1.0
3.42
1.22
1.12
1.34
1.24
8.2810
8.2632
8.0992
7.7261
7.7084
7.6631
7.5541
7.5380
7.4659
7.3113
7.1813
7.1670
7.1501
6.9952
6.9786
6.9586
6.9420
6.7498
6.7189
6.6957
6.6645
X : ppm : 1H

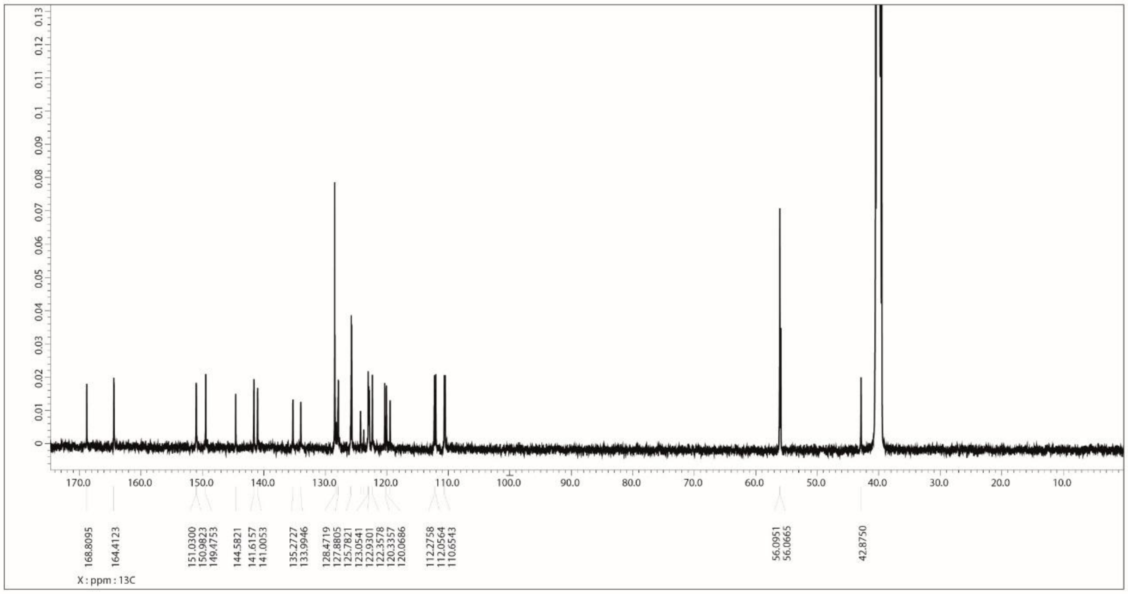

168.8095
164.4123
151.0300
150.9823
149.4753
144.5821
141.6157
141.0053
135.2727
133.9946
128.4719
127.8805
125.7821
123.0541
122.9301
122.3578
120.3357
120.0686
112.2758
112.0564
110.6543
56.0951
56.0665
42.8750
X : ppm : 13C

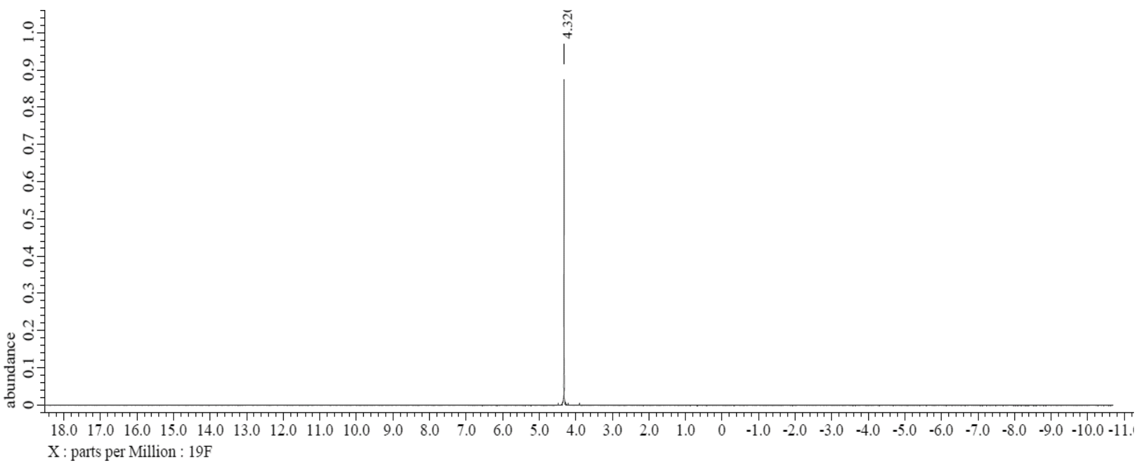

1.

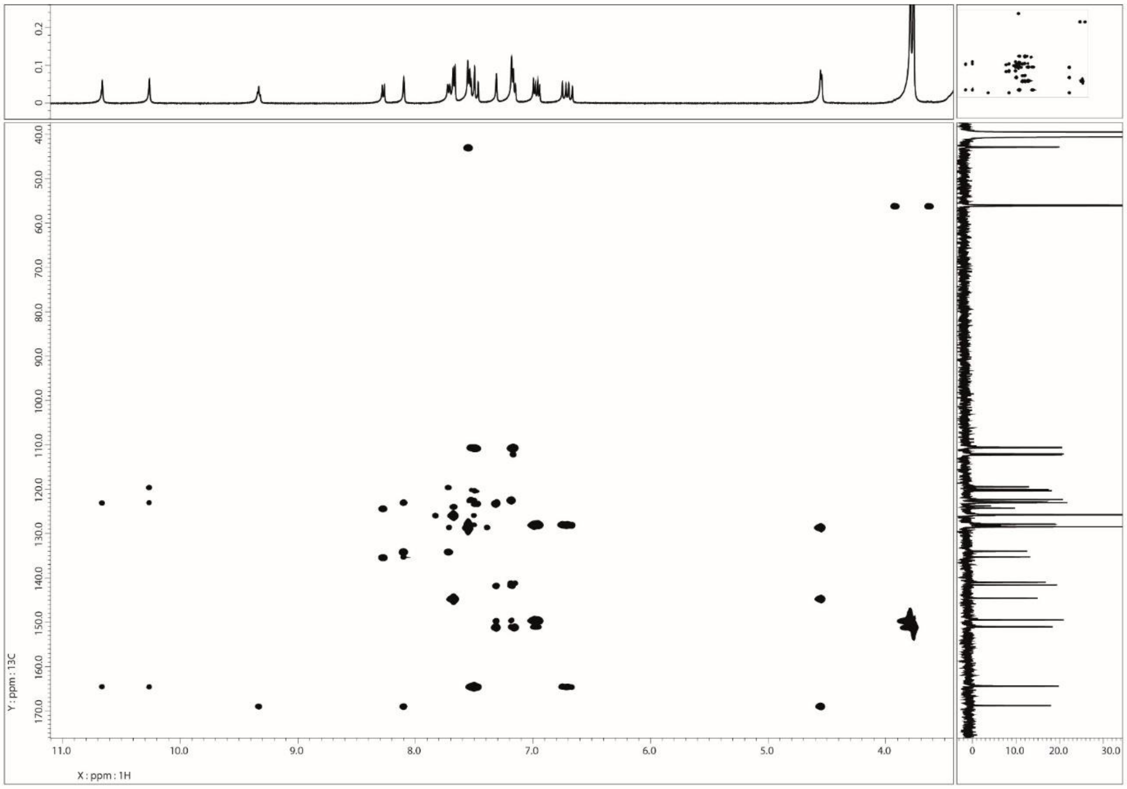

3.2 Compound **3**

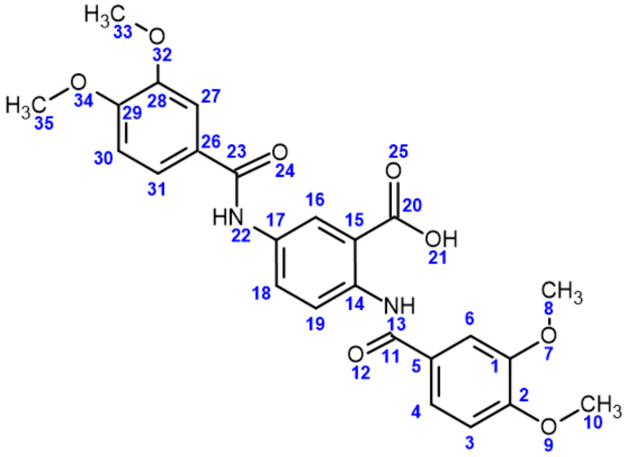

^1H NMR (DMSO-d_6, 500 MHz): δ = 12.23 (1H, s, H13), 10.18 (1H, s, H22), 8.62 (1H, d, J = 8.8 Hz, H19), 8.45 (1H, d, J = 2.5 Hz, H16), 7.94 (1H, dd, J = 8.8, 2.5 Hz, H18), 7.58 (1H, dd, J = 8.5, 1.8 Hz, H4), 7.51 (1H, d, J = 1.8 Hz, H6) 7.50 (1H, dd, J = 8.5, 1.8 Hz, H31), 7.45 (1H, d, J = 1.8 Hz, H27), 7.05 (1H, d, J = 8.5 Hz, H3), 7.01 (1H, d, J = 8.5 Hz, H30), 3.80 (6H, s, H10, H35), 3.79 (3H, s, H8*), 3.76 (3H, s, H32*), the assignment could be change. $^{13}C\{^1H\}$ NMR (DMSO-d_6, 125 MHz) δ: 170.60 C20), 165.49 (C23), 164.59 (C11), 152.39 (C2), 152.14 (C29), 149.10 (C1), 148.73 (C28), 137.60 (C14), 134.38 (C17), 127.29 (C5), 127.04 (C26), 126.57 (C18), 123.59 (C16), 121.62 (C4), 120.69 (C31), 120.30 (C19), 117.53 (C15), 111.70 (C3), 111.31 (C6, C30), 110.63 (C27), 56.18 (C10), 56.12 (C35), 56.09 (C8), 55.95 (C32).

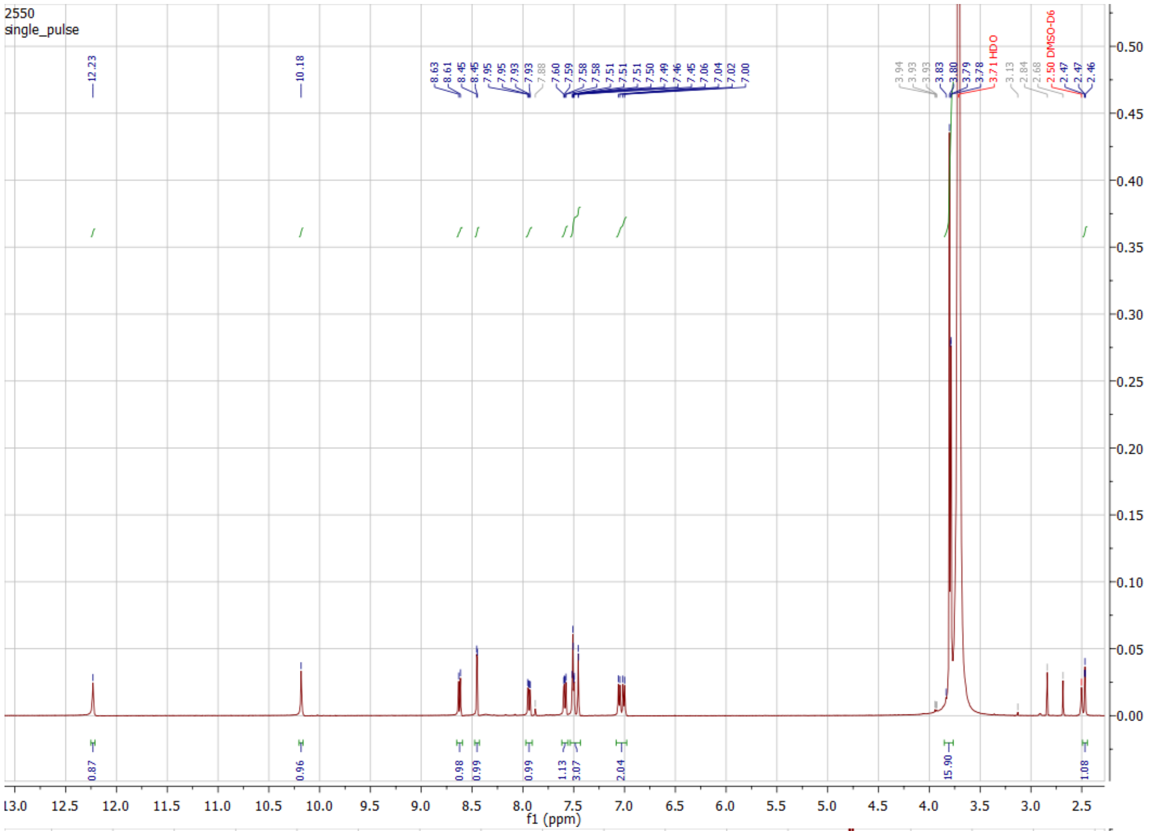
2550
single_pulse
f1 (ppm)

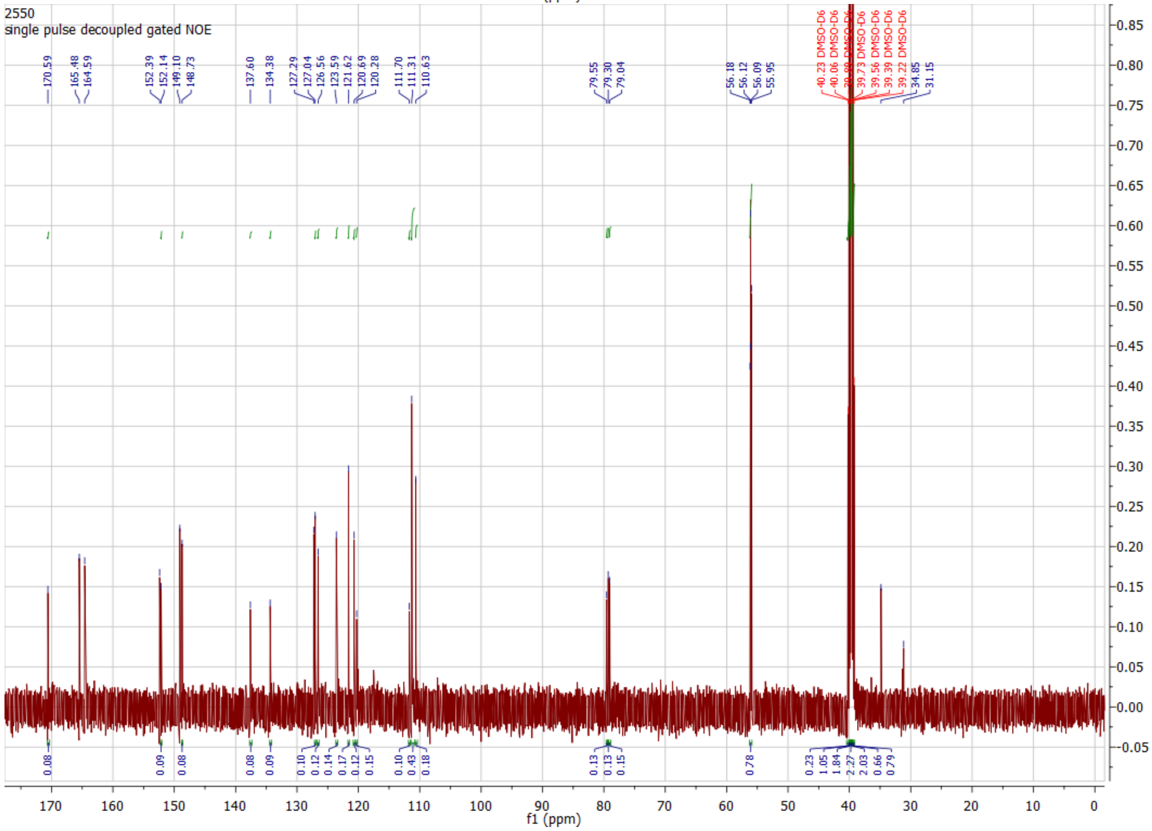
2550
single pulse decoupled gated NOE
f1 (ppm)

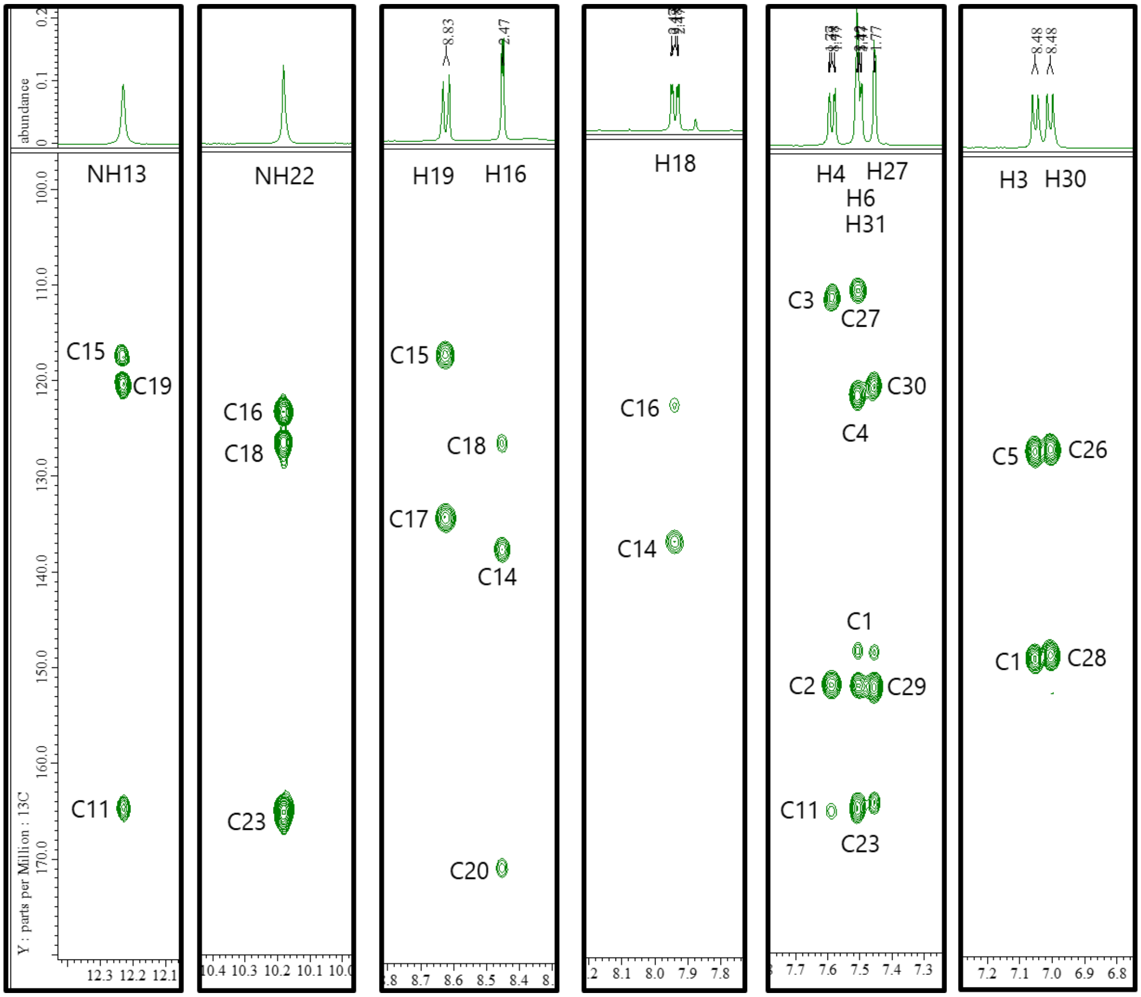

abundance
Y : parts per Million : 13C
NH13
C15
C19
C11
NH22
C16
C18
C23
H19
H16
C15
C18
C17
C14
C20
H18
C16
C14
H4
H27
H6
H31
C3
C27
C30
C4
C1
C2
C29
C11
C23
H3
H30
C5
C26
C1
C28

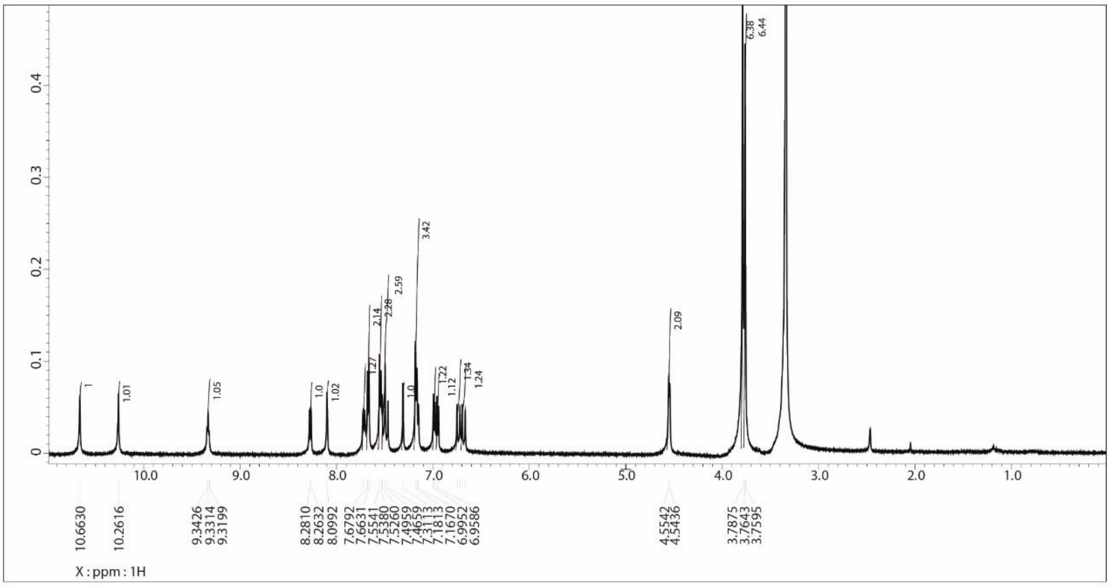
0.4
0.3
0.2
0.1
0
1
1.01
1.05
1.0
1.02
1.27
2.14
2.28
2.59
1.0
3.42
1.22
1.12
1.34
1.24
2.09
6.38
6.44
10.0
9.0
8.0
7.0
6.0
5.0
4.0
3.0
2.0
1.0
10.6630
10.2616
9.3426
9.3314
9.3199
8.2810
8.2632
8.0992
7.6792
7.6631
7.5541
7.5380
7.5260
7.4959
7.4659
7.3113
7.1813
7.1670
6.9952
6.9586
4.5542
4.5436
3.7875
3.7643
3.7595
X : ppm : 1H

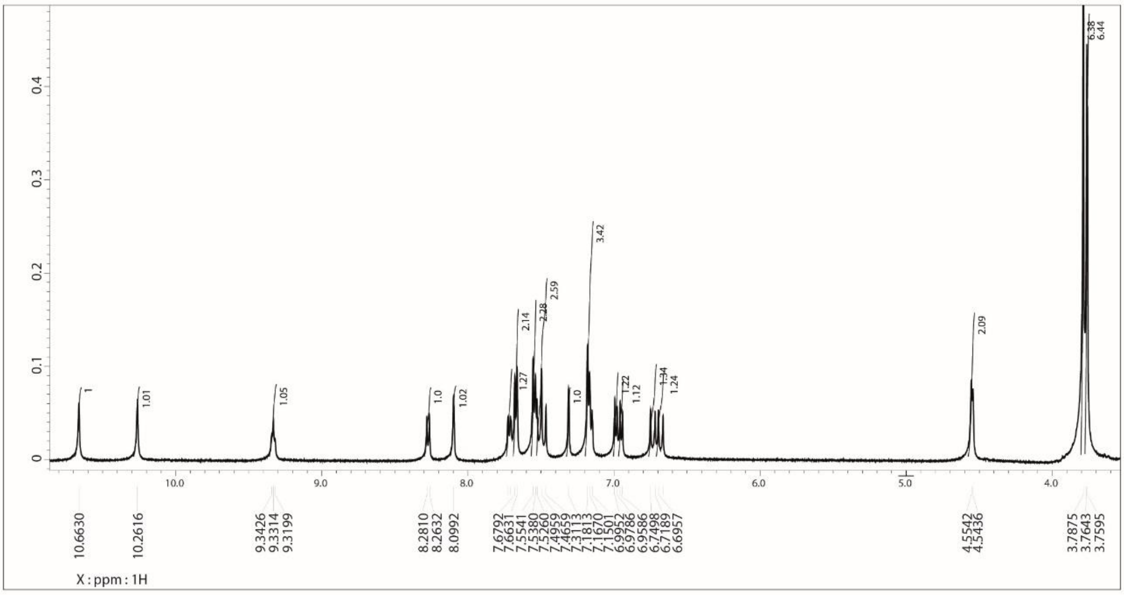
0.4
0.3
0.2
0.1
0
1
1.01
1.05
1.0
1.02
1.27
2.14
2.28
2.59
1.0
3.42
1.22
1.12
1.34
1.24
2.09
6.38
6.44
10.0
9.0
8.0
7.0
6.0
5.0
4.0
10.6630
10.2616
9.3426
9.3314
9.3199
8.2810
8.2632
8.0992
7.6792
7.6631
7.5541
7.5380
7.5260
7.4959
7.4659
7.3113
7.1813
7.1670
7.1501
6.9952
6.9786
6.9586
6.7498
6.7189
6.6957
4.5542
4.5436
3.7875
3.7643
3.7595
X : ppm : 1H

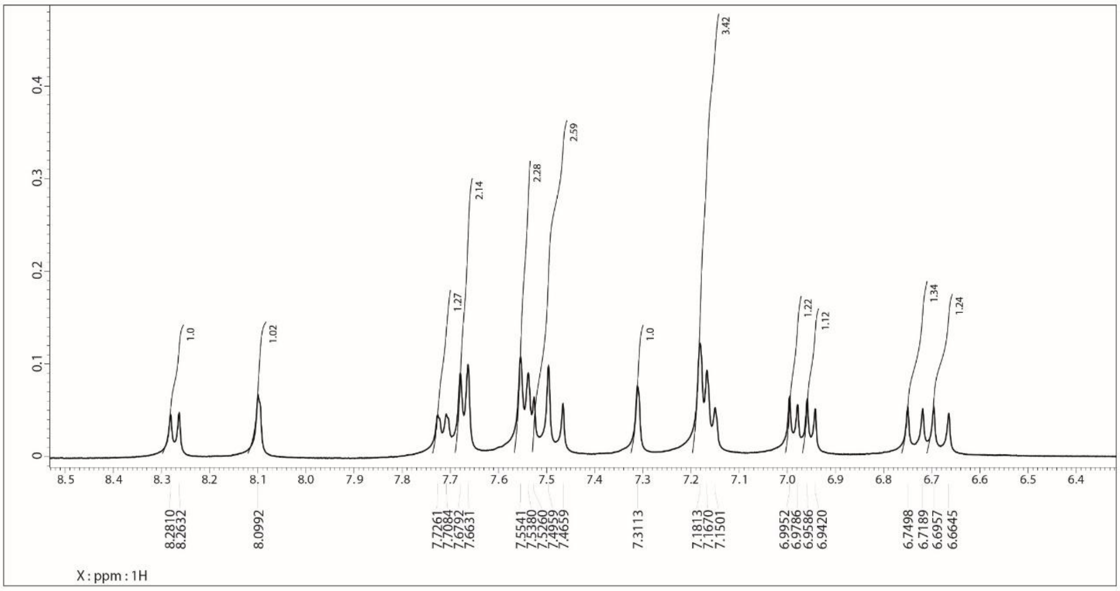
X : ppm : 1H

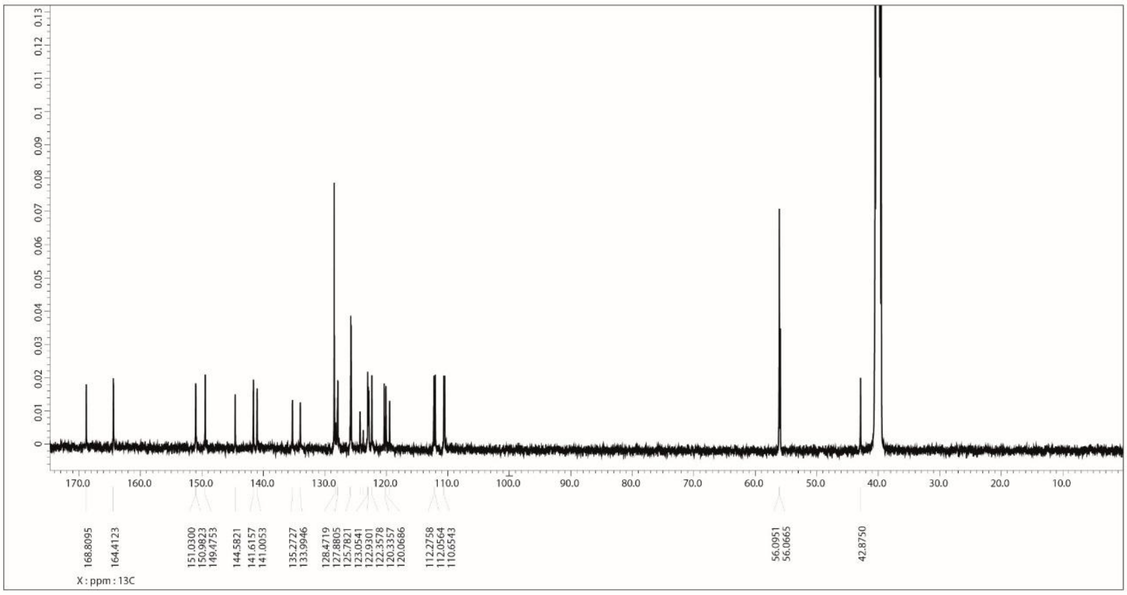
X : ppm : 13C

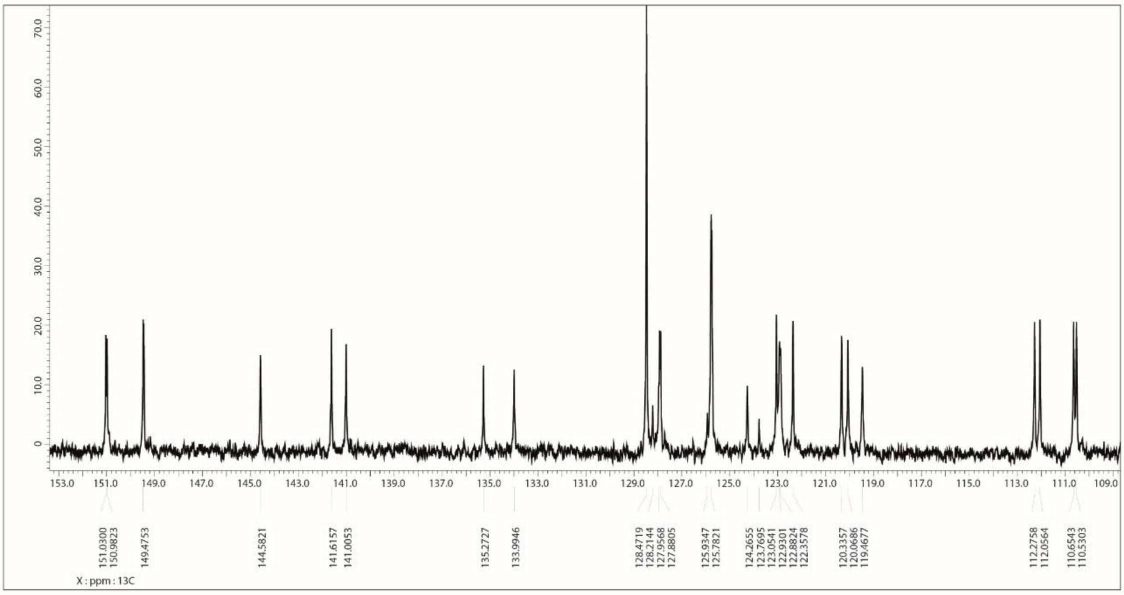
X : ppm : 13C

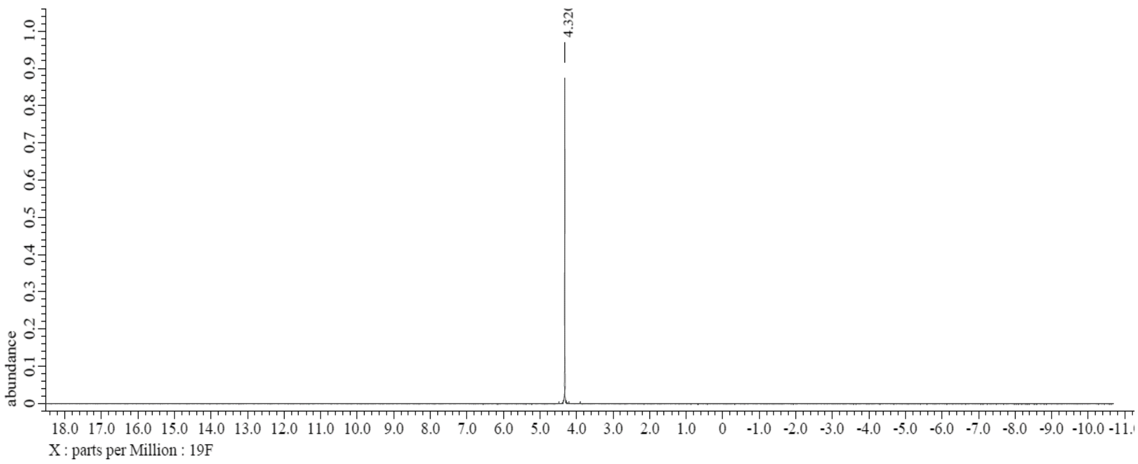

4.320
abundance
X : parts per Million : 19F

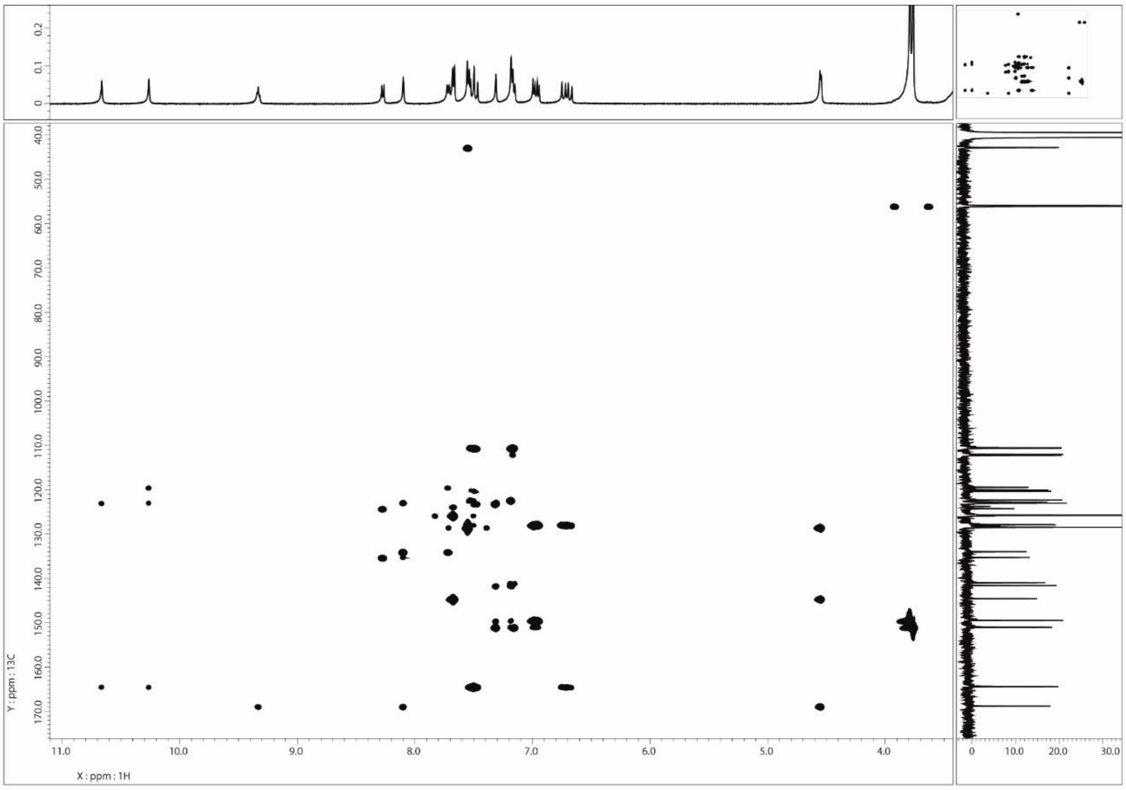

Y : ppm : 13C
X : ppm : 1H

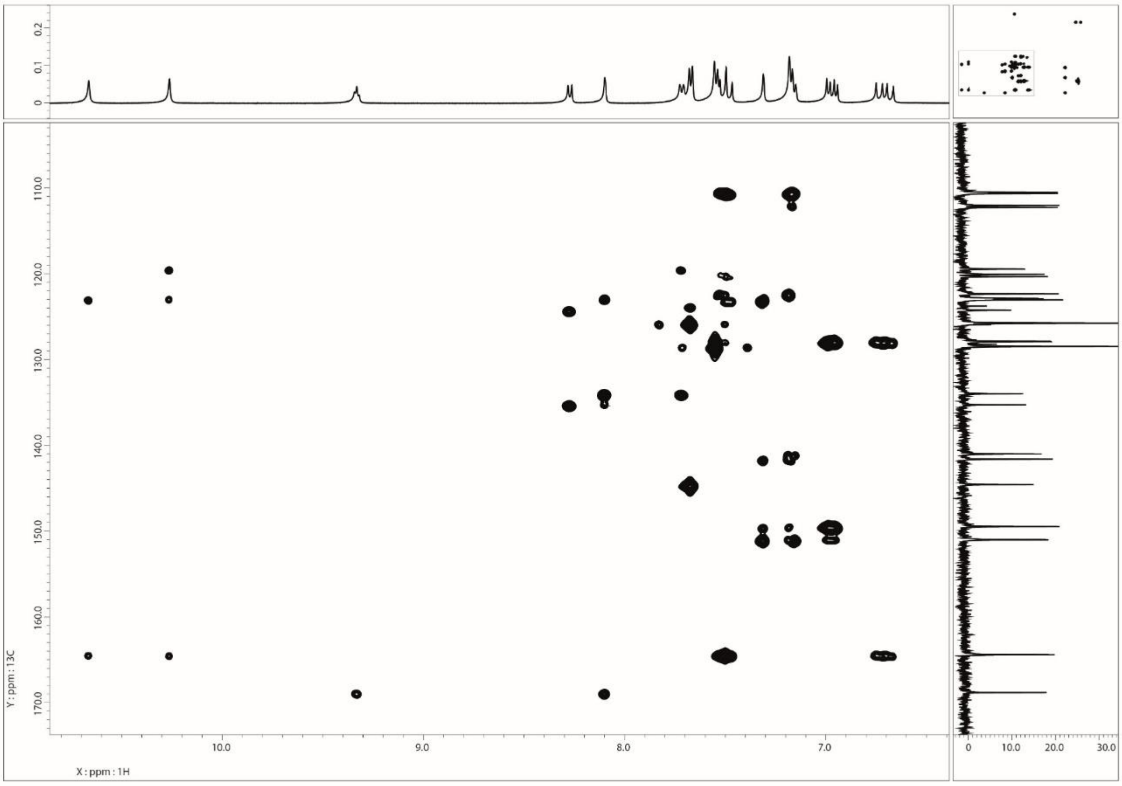
X : ppm : 1H
Y : ppm : 13C
10.0
9.0
8.0
7.0
110.0
120.0
130.0
140.0
150.0
160.0
170.0
0
0.1
0.2
10.0
20.0
30.0

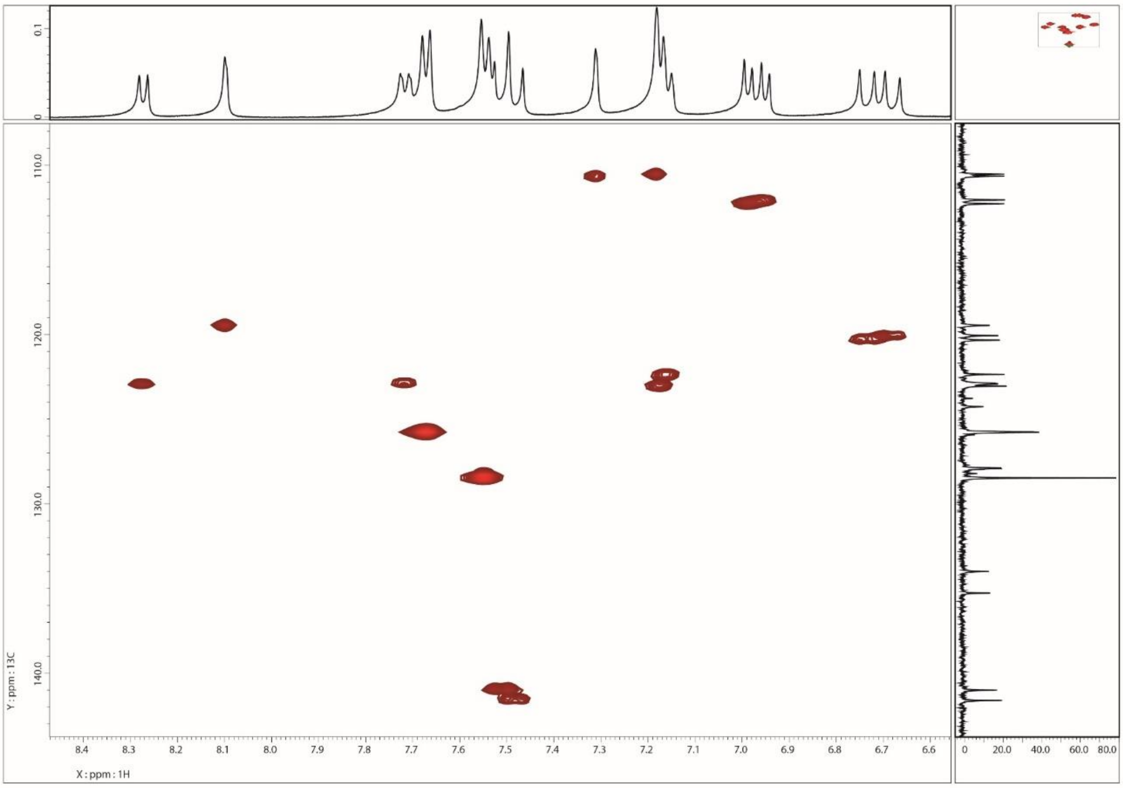
X : ppm : 1H
Y : ppm : 13C
8.4
8.3
8.2
8.1
8.0
7.9
7.8
7.7
7.6
7.5
7.4
7.3
7.2
7.1
7.0
6.9
6.8
6.7
6.6
110.0
120.0
130.0
140.0
0
0.1
20.0
40.0
60.0
80.0
